# Stable oncogenic silencing *in vivo* by programmable and targeted *de novo* DNA methylation in breast cancer

**DOI:** 10.1038/onc.2014.470

**Published:** 2015-02-16

**Authors:** S Stolzenburg, A S Beltran, T Swift-Scanlan, A G Rivenbark, R Rashwan, P Blancafort

**Affiliations:** 1 Cancer Epigenetics Group, The Harry Perkins Institute of Medical Research, Western Australia & School of Anatomy, Physiology and Human Biology, M309, The University of Western Australia, Nedlands, Western Australia, Australia; 2 Department of Pharmacology, School of Medicine, University of North Carolina at Chapel Hill, Chapel Hill, NC, USA; 3 Lineberger Comprehensive Cancer Center/School of Nursing, University of North Carolina, Chapel Hill, NC, USA; 4 Department of Pathology and Laboratory Medicine, University of North Carolina School of Medicine, Chapel Hill, NC, USA

## Abstract

With the recent comprehensive mapping of cancer genomes, there is now a need for functional approaches to edit the aberrant epigenetic state of key cancer drivers to reprogram the epi-pathology of the disease. In this study we utilized a programmable DNA-binding methyltransferase to induce targeted incorporation of DNA methylation (DNAme) in the *SOX2* oncogene in breast cancer through a six zinc finger (ZF) protein linked to DNA methyltransferase 3A (ZF-DNMT3A). We demonstrated long-lasting oncogenic repression, which was maintained even after suppression of ZF-DNMT3A expression in tumor cells. The *de novo* DNAme was faithfully propagated and maintained through cell generations even after the suppression of the expression of the chimeric methyltransferase in the tumor cells. Xenograft studies in NUDE mice demonstrated stable *SOX2* repression and long-term breast tumor growth inhibition, which lasted for >100 days post implantation of the tumor cells in mice. This was accompanied with a faithful maintenance of DNAme in the breast cancer implants. In contrast, downregulation of *SOX2* by ZF domains engineered with the Krueppel-associated box repressor domain resulted in a transient and reversible suppression of oncogenic gene expression. Our results indicated that targeted *de novo* DNAme of the *SOX2* oncogenic promoter was sufficient to induce long-lasting epigenetic silencing, which was not only maintained during cell division but also significantly delayed the tumorigenic phenotype of cancer cells *in vivo,* even in the absence of treatment. Here, we outline a genome-based targeting approach to long-lasting tumor growth inhibition with potential applicability to many other oncogenic drivers that are currently refractory to drug design.

## Introduction

Cytosine methylation in mammalian DNA is regarded as a key epigenetic modification controlling essential processes such as imprinting, silencing of retrotransposons and cell differentiation.^1^ In addition to DNA methylation (DNAme), repressive histone post-translational modifications reinforce gene inactivation, resulting in the formation of inactive chromatin, namely heterochromatin.^2^ Importantly, the epigenetic information must be stably transmitted during mitosis for the proper maintenance of cellular identity, a process referred as epigenetic memory.^3^ In pathological states such as cancer, the epigenetic landscape of normal cells becomes disrupted. Aberrant incorporation or removal of DNAme in cancer cells leads to genome-wide alteration of gene expression, including inactivation of tumor suppressor genes and reactivation of oncogenes.^4^ Notably, changes in DNAme patterns are characteristic hallmarks of the distinct intrinsic subtypes of breast cancer. Poorly differentiated, highly proliferative basal-like breast cancers associated with stem/progenitor cell-like features are significantly hypomethylated relative to the other breast cancer subtypes. Importantly, these tumors are driven by aberrant activation of multiple developmental transcription factors (TFs), which fuel the tumor with sustained proliferation, drug resistance and metastatic capacity.^5, 6, 7^

The High Mobility Group oncogenic TF *SOX2* is normally expressed in embryonic stem cells and neural progenitor cells, where it maintains self-renewal.^8, 9^ DNAme in the *SOX2* promoter and enhancer regions functions as an epigenetic switch, which forces cells to activate multiple differentiation pathways.^10^ *SOX2* is therefore not expressed in most normal adult tissues.^10, 11, 12^ Moreover, aberrant reactivation of *SOX2* has been detected in ~43% of basal-like breast cancers and in several other malignancies, including glioblastoma, lung, skin, prostate and ovarian carcinomas.^13^ *SOX2* overexpression in tumor specimens has been associated with both promoter hypomethylation relative to adjacent normal tissue and copy number amplifications.^14, 15^ The overexpression of *SOX2* in breast cancer has been shown to directly activate *CYCLIN D1*, resulting in an increased mitotic index and proliferation.^16, 17^ The downregulation of *SOX2* by RNA interference decreased the tumorigenic phenotype in the lung, breast and ovarian cancers.^13, 16, 18^ However, a major limitation of small interferin RNA and small hairpin RNA approaches in cancer therapy has been the short half-life of small RNA or the methylation of the virally encoded small hairpin RNA promoter, which results in transient effects *in vivo*.

In light of the essential role of DNAme in the regulation of *SOX2* expression we hypothesized that targeted *de novo* methylation in the *SOX2* promoter would result in an epigenetic 'off' switch, forcing cancer cells to undertake differentiation programs. Furthermore, because DNAme is read and written by endogenous proteins and faithfully transmitted during cell division, we reasoned that artificial incorporation of *de novo* DNAme in the *SOX2* promoter would confer stable oncogenic silencing, resulting in a sustained blockade of cell growth, faithfully propagated in successive cell generations. The forced epigenetic reprogramming of an oncogene toward a 'normal-like' state, which will be stably transmitted through mitosis, remains a hitherto unexplored paradigm in cancer research; the resulting therapeutic effect would provide durability and sustained anticancer response (memory).

Herein we took advantage of previously characterized artificial TFs targeting the *SOX2* promoter made of six zinc finger (6ZF) domains, recognizing 18 base pair (bp) sites in the core promoter.^17^ These 6ZFs were linked to the catalytic domain of DNA methyltransferase 3A (DNMT3A), an enzyme that catalyzes *de novo* DNAme, to correct the aberrant methylation state of *SOX2* in cancer cells. We show that targeted DNAme is sufficient to initiate, and even reinforce, potent and mitotically heritable silencing of *SOX2* expression. Our results indicate that the engineering of artificial DNA-binding domains (DBDs) linked to DNMT3A can be utilized to promote long-lasting *SOX2* silencing and represents a promising therapeutic approach to minimize tumor relapse. Our approach could be used to promote customizable heterochromatization and epigenetic silencing of elusive cancer drivers mapped by recent genomic projects, such as TFs and small GTPAses (*RAS*), for which no drug is currently available.

## Results

### ZF598-DNMT3A initiates *SOX2* downregulation accompanied by stable inhibition of cancer cell growth

**Figure 1:**
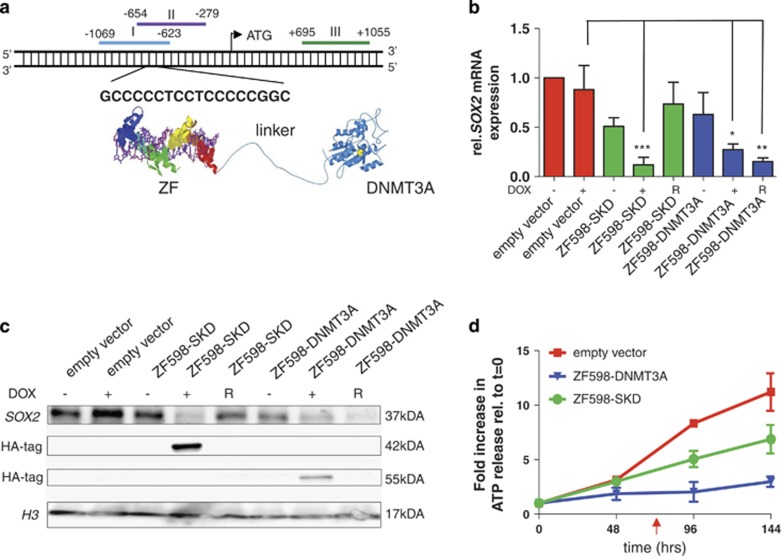
Stable downregulation of the SOX2 expression by ZF598-DNMT3A. (**a**) Schematic illustration of the *SOX2* promoter indicating the domain structure of the ZF598-DNMT3A construct, its binding site and the three amplicons analyzed by sodium bisulfite sequencing or MassARRAYs (amplicon I, blue; II, purple and III, green). (**b**) Quantification of *SOX2* mRNA expression by qRT-PCR in MCF7 cells. Cells were stably transduced with empty vector, ZF598-SKD and ZF598-DNMT3A. The expression of the ZF fusion was controlled by addition or removal of doxycycline (Dox); R=Dox removal. Cells were induced with Dox every 48 h and either harvested at 72 h after first induction (+Dox) or removed from Dox and subcultured for 8 days and processed for western blot. Error bars represent standard deviation (s.d.) (***P< 0.001, **P<0.01, *P<0.05). (**c**) Detection of SOX2 by western blot. The C-terminal Hemagglutinin (HA) tag was used for immunodetection of the ZF proteins. An anti-histone H3 antibody was used as loading control. (**d**) Cell viability of MCF7 cells assessed by Cell TiterGlo assays. Empty vector, ZF598-SKD and ZF598-DNMT3A-transduced cells were induced with Dox for 48 h and removed from Dox after 72 h (red arrow). *P-*values between empty vector and ZF598-DNMT3A and between ZF598-SKD and ZF598-DNMT3A were *P*<0.001 and *P*<0.05 respectively, at 144 h.

To induce targeted DNAme in the *SOX2* locus we took advantage of our previously characterized 6ZF domains designed to recognize 18 bps sequences in the *SOX2* proximal promoter (Figure 1a).^17^ Subsequently, we engineered the catalytic domain of the human DNMT3A to the C terminus of the 6ZF domains and in frame with a 13 amino-acid flexible linker, to produce the ZF598-DNMT3A (Figure 1a) and the ZF552-DNMT3A (Supplementary Figure S1) constructs.^19^ The same 6ZF arrays linked to the repressor super Krueppel-associated box domain (SKD, ZF598-SKD) were used as a control, as this domain promotes potent KAP1-dependent repression when linked at the N terminus of the 6ZFs, without induction of DNAme.^17, 19^

**Figure 2:**
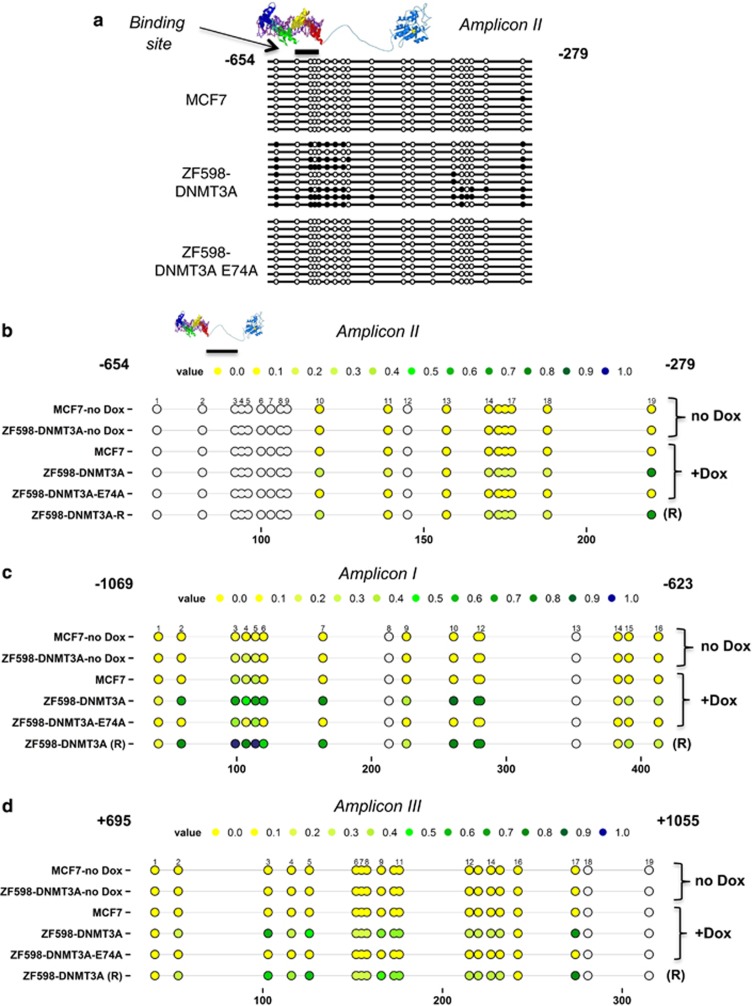
Expression of the ZF598-DNMT3A induces targeted DNA methylation in the *SOX2* promoter. (**a**) Sodium bisulfite-sequencing analysis of DNA derived from MCF7 cells stably transduced with empty vector, ZF598-DNMT3A and ZF598-DNMT3A-E74A mutant and induced with Dox. The analyzed amplicon II expands from −654 to −279 base pairs (bps) upstream the translation start site and includes the 6ZF-binding site (position −598 relative to the translation start site). Gray circles indicate not analyzed methylation values owing to CpGs with high- or low-mass Dalton peaks falling outside the conservative window of reliable detection for the EpiTYPER software, colored circles indicate variously methylated CpGs. (**b**) MassARRAY analysis of the same amplicon as in (**a**). Circles indicate the CpG dinucleotides in the amplicon (Color code: yellow=unmethylated CpG to blue=100% methylated CpG). The percentage of meCpG was determined in MCF7 control-transduced cells and in MCF7 cells stably transduced with ZF598-DNMT3A or ZF-598-DNMT3A-E74A in absence of Dox (no Dox), presence of Dox (+Dox) or after Dox removal (R, eight days). (**c**) MassARRAY analysis of amplicon I (−1069 to −623 bps upstream of the translation start site) in Dox-free conditions (no Dox) and after Dox induction (+Dox) Dox removal (R). (**d**) MassARRAY analysis of amplicon III (+695 to +1055 bps) downstream of the translation start site, same conditions as above.

We chose the MCF7 breast cancer cells as a model cell line to study the temporal dynamics of the incorporation of DNAme, as these cells express high levels of SOX2, and the promoter does not contain methylated CpG dinucleotides (Figure 2a). The 6ZF constructs were delivered into MCF7 cells using inducible retroviral vectors^17^ and stable clones were isolated in which the temporal expression of the ZF598-DNMT3A and ZF598-SKD fusions was controlled by doxycycline (Dox) treatment.

Upon induction of the ZF598-DNMT3A and ZF598-SKD expression with Dox (+Dox), *SOX2* mRNA levels decreased by 90% and 73%, respectively, as compared with control (empty vector) transduced cells (Figure 1b). When Dox was removed for up to 8 days (~10 cell generations) from the culture media (R, Figures 1b and c) cells transduced with ZF598-SKD restored *SOX2* expression to levels that were similar to those of uninduced cells, whereas cells expressing the ZF598-DNMT3A showed a persistent decrease of *SOX2* expression (85% relative to uninduced cells) upon Dox removal (Figure 1b). These findings suggested that the ZF598-DNMT3A construct conferred transcriptional memory in the targeted *SOX2* locus. The *SOX2* transcriptional silencing induced by ZF598-DNMT3A was accompanied by a significant decrease in SOX2 protein expression. This suppression was sustained in Dox-removal conditions in the absence of expression of the ZF598-DNMT3A, and even resulted in increased levels of SOX2 silencing relative to +Dox cells as demonstrated by western blot (Figure 1c).

We next investigated whether the ZF598-DNMT3A fusion was able to maintain suppression of tumor cell growth in Dox-removal conditions. We monitored changes in cell viability over time associated with induction and subsequent removal of the ZF protein expression (Figure 1d). MCF7 cells stably transduced with either empty vector control, ZF598-SKD or ZF598-DNMT3A, were treated for 72 h with Dox to induce the expression of the ZF proteins. Next, Dox was removed from the culture media (Figure 1d, red arrow) and cell viability was monitored for the next 144 h. Cells expressing empty vector or ZF598-SKD had significantly higher proliferation rates upon Dox removal as compared with ZF598-DNMT3A transduced cells, which retained a more robust inhibition of cell proliferation after Dox removal. These results demonstrate the unique capacity of the 6ZF-DNMT3A fusions to establish an oncogenic silencing state at both the mRNA and protein level that was stably maintained through cell generations.

### ZF598-DNMT3A silences *SOX2* by initiation and faithful propagation of DNAme

We subsequently investigated whether ZF598-DNMT3A was able to catalyze targeted *de novo* DNAme in the *SOX2* promoter. MCF7 cells stably transduced with ZF598-DNMT3A were treated with Dox to induce the expression of the ZF598-DNMT3A. Next, cells were either harvested at 72 h or removed from Dox and passaged for 8 additional days in Dox-free conditions. These Dox-removal conditions completely suppressed the expression of the ZF protein, as determined by western blotting of nuclear extracts using an anti-HA antibody (Figure 1c).

Conventional sodium bisulfite sequencing was first conducted to quantify the incorporation of DNAme in the amplicon containing the ZF598-DNMT3A binding site (Amplicon II, Figure 1a). Dox-induced expression of ZF598-DNMT3A resulted in an increase of DNAme of up to 90% at specific CpG dinucleotides (Figure 2a). The incorporation of DNAme was also validated by MassARRAY analysis (Figures 2b and d). Control cells or cells expressing the catalytic mutant ZF598-DNMT3A-E74A revealed no increase of CpG methylation, demonstrating that the induction of DNAme required the catalytic activity linked to the ZF598. Similar results were obtained utilizing a second ZF protein (ZF552-DNMT3A) targeting an 18 bps region 552 bps upstream of the translation start site (Supplementary Figure S1), which demonstrates that the approach is generalizable for other DBDs targeting *SOX2*. Furthermore, ectopic overexpression of the catalytic domain of DNMT3A (untargeted DNMT3A) lacking the SOX2-specific DBDs resulted in an unspecific background incorporation of DNAme (Supplementary Figure S1). The untargeted construct was not capable of downregulating *SOX2* messenger RNA levels or to induce proliferative arrest. Furthermore, when the DNMT3A was linked to 6ZF DBDs specific for the *MASPIN* tumor suppressor gene context, instead of an oncogenic context, targeted methylation was observed in the *MASPIN* promoter accompanied by an increase in cell proliferation.^19^ Reciprocally, no changes in DNAme were observed in the *MASPIN* promoter context with the SOX2-specific DBDs linked to DNMT3A (Supplementary Figure S2). These results indicated that the approach required two functional components, the targeting DBD and a catalytically active DNMT3A domain.

Importantly, the *de novo* pattern of CpG methylation initiated by ZF598-DNMT3A in the *SOX2* promoter was stably and faithfully maintained over several cell generations *in vitro*, even 8 days after Dox removal (Figure 2b) when the expression of the targeted methyltransferase was not longer detectable by immunoblotting. Furthermore, both the pattern and intensity of the induced methylation (40–90% depending on the specific CpG nucleotide) were maintained over the time course of the experiment.

To evaluate the spreading of DNAme in the *SOX2* locus after the initial induction, we analyzed two additional amplicons (amplicon I and III) flanking the 6ZF-binding site, encompassing ~1 kbps up- and downstream of the translation start site (Figures 2c and d). Sequencing analysis of amplicon I (−1069 to −623 bps upstream of the translation start site) demonstrated an increase of DNAme upon ZF598-DNMT3A expression as compared with the MCF7 empty vector transduced cell line or ZF598-DNMT3A-E74A-transduced cells. The frequencies of DNAme in amplicon I were significantly higher than those immediately adjacent to the ZF-binding site, suggesting that DNAme was possibly spread and even reinforced in the flanking regions. Most importantly, after discontinuation of the ZF598-DNMT3A expression (Dox removal), the *de novo* methylation additionally increased up to 97% at specific CpG sites in Amplicon I (Figure 1c).

As last, the MassARRAY analysis of DNAme in amplicon III (+695 to +1055 bps downstream of the translation start site, Figure 2d) revealed no methylation in MCF7 control and ZF598-DNMT3A cells in the absence of Dox. Upon ZF598-DNMT3A expression (+Dox) CpG methylation increased up to 80%, and no DNAme was induced in cells expressing the ZF598-DNMT3A-E74A mutant. Our time-course analysis suggests that the *de novo* DNAme induced by the artificial methyltransferase results in both, a phase of induction of gene silencing upon Dox induction, and a phase of maintenance and reinforcement of silencing (Dox removal), which could be associated with propagation or spreading of DNAme further away from the 6ZF site during DNA replication. These results further support the increased downregulation of *SOX2* expression after Dox removal previously observed by quantitative reverse transcription polymerase chain reaction and western blot analysis and outline the importance of CpG dinucleotides flanking the core promoter for the regulation of *SOX2* expression.

### ZF598-DNMT3A expression confers anticancer memory and reduces tumor growth in a breast cancer xenograft in NUDE mice

**Figure 3:**
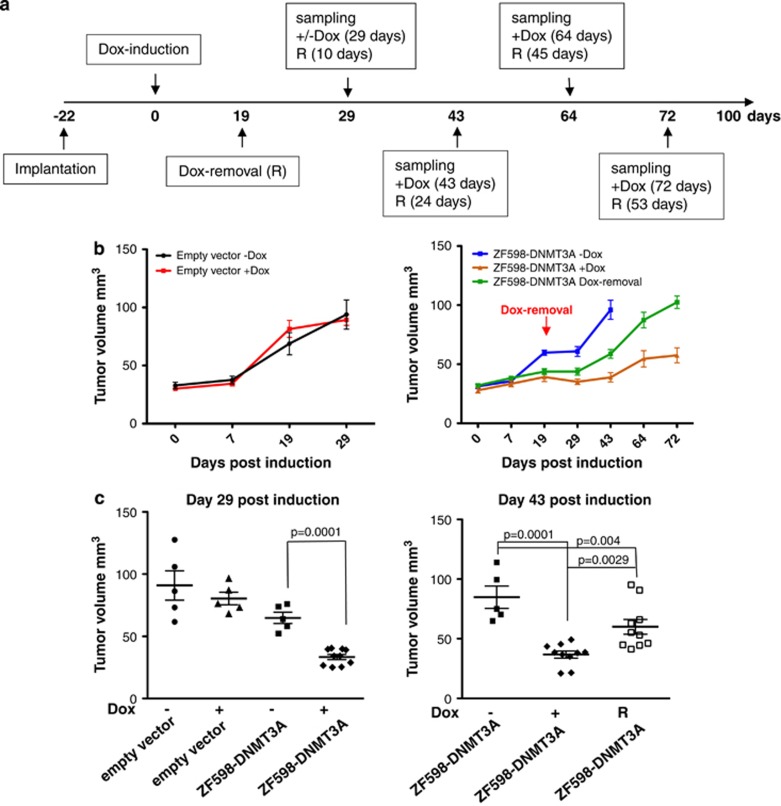
The *de novo* DNA methylation and oncogenic silencing induced by ZF598-DNMT3A are maintained long term in a xenograft model of breast cancer. (**a**) Timeline of the subcutaneous tumor injections of MCF7 cells in NUDE mice. (**b**) Time course plot monitoring tumor volumes of empty vector control and ZF598-DNMT3A animals induced (+Dox) and uninduced (−Dox). Left panel: tumor growth of empty vector control animals. Right panel: tumor volumes of animals implanted with ZF598-DNMT3A. (**c**) Left panel: tumor volumes of ZF598-DNMT3A and control animals at day 29 post induction. Right panel: tumor volumes of ZF598-DNMT3A implanted animals at day 43, when the −Dox control tumors were collected. *P-*values between groups are indicated.

To analyze whether the ZF598-DNMT3A construct induced phenotypic memory *in vivo* we took advantage of our inducible MCF7 cell lines stably transduced with either the catalytically active ZF598-DNMT3A or the empty vector control. A total of 2 × 10^6^ MCF7 cells transduced with either ZF598-DNMT3A or control were implanted into the flank of NUDE mice and allowed to grow for 22 days before switching the animals to a Dox-containing diet (Figure 3a). Within each group *N*=5 animals were maintained in a Dox-free diet. At day 19 post induction, half of the ZF598-DNMT3A injected animals from the +Dox group were removed from the Dox diet (*N*=10), to withdraw the expression of the ZF methyltransferase (R). In contrast with ZF598-DNMT3A, catalytic dead ZF598-DNMT3A-E74A mutant cells failed to reduce MCF7 cell viability *in vitro* and thereby were not further injected in mice (Supplementary Figure S3).

Over the period of 43 days, a significant inhibition (*P*=0.0001) of tumor growth was detected in animals injected with ZF598-DNMT3A receiving a Dox-containing diet (Figure 3b, right panel), which was superior to the inhibition we have reported for Krueppel-associated box-containing repressors.^17^ Furthermore, this reduction of tumor growth was maintained up to 72 days after Dox induction (*P*=0.001). Importantly, two animals induced with the ZF598-DNMT3A construct had completely regressed tumor burden and could not be detected by caliper measurements. As expected, no significant change in tumor growth was detected in animals injected with empty vector (Figures 3b and c, left panel).

To study the stability of the therapeutic effect we removed the Dox from the diet of half (*N*=10) of the ZF598-DNMT3A animals (Dox-removal group), while maintaining the other half under +Dox conditions. These groups allowed us to investigate whether the tumor growth inhibition mediated by *SOX2* methylation was stably transmitted even after removal of ZF598-DNMT3A expression. Tumor volumes in the Dox-free, Dox-induced and Dox-removal groups were monitored for 43 days post induction, equal to 24 days post removal of Dox for the Dox-removal group (Figure 3c, right panel). The tumor volumes of ZF598-DNMT3A +Dox animals demonstrated a significant inhibition relative to ZF598-DNMT3A −Dox animals (*P*=0.0001). Furthermore, mice removed from a Dox diet maintained a significant reduction of tumor burden (*P*=0.004) relative to uninduced animals (Figure 3c, right panel). It should be noted that the tumor sizes of mice removed from Dox slowly increased over time when compared with animals continuously placed under +Dox conditions. However, this effect could be due to the positive selection of cells carrying low levels of methylation in the xenografts or by functional compensation by another oncogenic driver.

**Figure 4:**
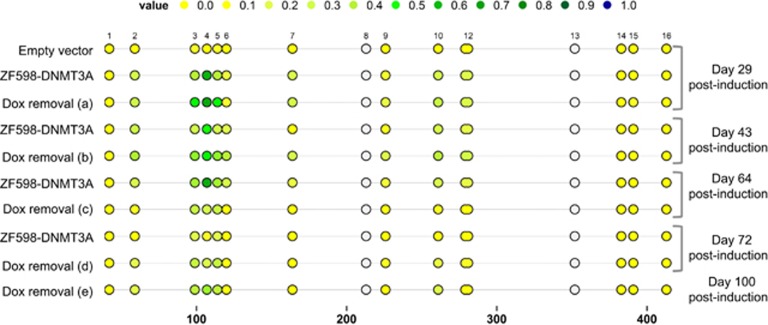
MassARRAY analysis of tumor DNA shows targeted induction and maintenance of DNA methylation. DNA from ZF598-DNMT3A +Dox and Dox-removal tumors was extracted at the indicated time points and subjected to sodium bisulfite conversion, followed by MassARRAY to detect DNA methylation frequencies. The amplicon I is located −1069 to −623 bps upstream of the translation start site. Each circle represents a CpG dinucleotide. Color code: yellow=unmethylated CpG, blue=100% methylated CpG. Gray circles indicate not analyzed methylation values due to CpGs with high or low mass Dalton peaks falling outside the conservative window of reliable detection for the EpiTYPER software.

To verify that the reduction of tumor growth in the ZF598-DNMT3A +Dox animals was associated with the incorporation of DNAme, MassARRAY analysis of tumor DNA was performed at different time points post induction (Figure 4). DNA samples extracted from empty vector +Dox tumors at 29 days post induction were used as control. Upon induction of ZF598-DNMT3A expression an increase of DNAme was detected at all time points of sampling relative to control. The comparison of meCpG frequencies in the *SOX2* amplicon I at day 43 post induction indicated that the majority of CpG dinucleotides were significantly more methylated in the ZF598-DNMT3A +Dox and Dox-removal tumors over the ZF598-DNMT3A −Dox tumors sampled in the experiment (Supplementary Figure S4 and Supplementary Table S1). Importantly, this methylation was maintained *in vivo* for more than 50 days after Dox removal in all samples analyzed. These data clearly demonstrated that the targeted DNAme was associated with tumor growth inhibition and with a significant decrease in the rate of tumor relapse when the treatment was discontinued.

### Breast tumor growth inhibition was maintained upon clearance of ZF598-DNMT3A expression

**Figure 5:**
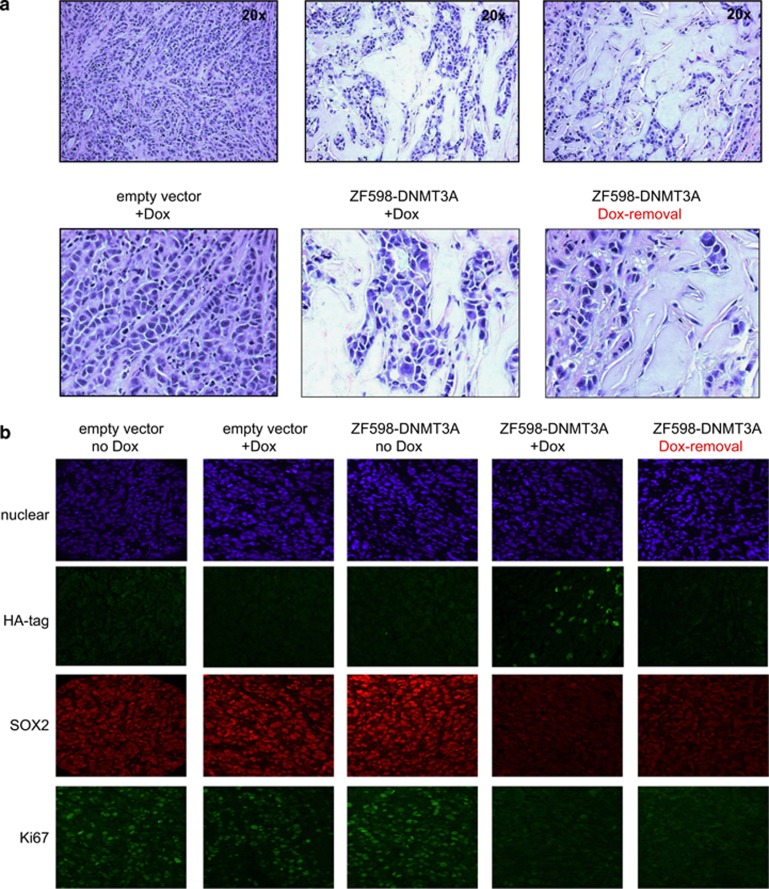
Histological and immunofluorescence analyses of tumor sections reveals phenotypic memory. (**a**) Hematoxylin and Eosin stains of representative ZF598-DNMT3A −Dox, +Dox and Dox-removal tumor sections. Sections of empty vector tumors were extracted 29 days post induction, sections of ZF598-DNMT3A +Dox were sampled after 54 days of Dox induction, and those of Dox-removal samples were harvested 45 days after Dox removal. Pictures were taken at × 20 and a detail of the image is shown. (**b**) Immunofluorescence on sections of empty vector and ZF598-DNMT3A tumors collected 29 days post induction. The expression of ZF598-DNMT3A (HA-tag, green), SOX2 (red) and the proliferation marker Ki-67 (green, bottom) in +Dox, −Dox and Dox-removal conditions are indicated. Images are taken at × 40 magnification.

To examine changes in the tumor morphology, Hematoxylin and Eosin staining was performed on sections of empty vector control and ZF598-DNMT3A tumors. The control tumors were collected 29 days post induction, the ZF598-DNMT3A tumors were continuously induced with Dox for 72 days and for the ZF598-DNMT3A Dox-removal tumors, Dox was withdrawn for 53 days after an initial induction of 19 days. Histological analysis of empty vector +Dox tumor sections revealed a high density of closely packed tumor cells at day 29 post induction (Figure 5a, left panel). In contrast, the ZF598-DNMT3A +Dox tumors exhibited a very striking change in the tumor architecture, with a more organized structure consisting of islands of tumor cells and an increase of intervening stroma, a phenotype that was maintained after removal of ZF598-DNMT3A expression (Figure 5a). In the analyzed tumors, this change in the tumor phenotype was accompanied with the significant downregulation of some mesenchymal markers, such as *SOX2*, *TWIST1* and *Vimentin* and a significant upregulation of some epithelial junction proteins such as *Claudin 4* as assessed by quantitative reverse transcription polymerase chain reaction (Supplementary Figure S5).

Immunofluorescence analysis of tumor sections revealed nuclear expression of the ZF598-DNMT3A protein in the +Dox group, but no observable signal in ZF598-DNMT3A −Dox animals (Figure 5b). This induction of methyltransferase expression correlated with a significant decrease in SOX2 expression in the tumors that received Dox, which was not observed in control (empty vector ±Dox) and ZF598-DNMT3A −Dox tumors. After removal of Dox, the ZF598-DNMT3A expression was not longer detected in the tumor sections. Importantly, a decrease of SOX2 expression was stably maintained *in vivo* after Dox removal relative to control or −Dox tumors. In addition, the downregulation of SOX2 expression correlated with a decrease in tumor cell proliferation, as indicated by the Ki-67 marker, which was maintained downregulated relative to uninduced cells even after removal of ZF598-DNMT3A expression for 10 days (Figure 5b). These results suggest that the *de novo* methylation patterns were robustly maintained during somatic cell division *in vivo* resulting in a remarkable phenotypic change of the tumor cells, suggesting loss of some mesenchymal features and gain of some epithelial-like features.

## Discussion

Promoter CpG methylation has an important role in controlling gene transcription and therefore contributes to the regulation of many biological processes. In cancer, aberrant DNAme is associated with initiation and progression of malignant disease. Like TFs, many oncogenic drivers in cancer are of undruggable nature, such as the small GTPases *KRAS* and *HRAS*. Therefore, the ability to program a heritable targeted silencing state in such major oncogenic drivers would be a high impact accomplishment with far reaching clinical implications for cancer treatment.

Targeted DNAme by designer ZFs linked to the catalytic domain of DNA methyltransferases has been recently demonstrated by our group and others.^19, 20, 21, 22, 23, 24, 25^ We have previously shown that engineered 6ZFs binding the *MASPIN* tumor suppressor promoter linked to DNMT3A induced targeted DNAme in the promoter, and that the resulting phenotypic effect of these proteins was the activation of cancer cell growth.^19^ Here, we demonstrated that DNAme targeted to the *SOX2* oncogenic promoter via 6ZF domains linked to DNMT3A was associated with a significant inhibition of tumor growth in a xenograft mouse model of breast cancer. We took advantage of two different 6ZF proteins engineered to bind *SOX2* promoter (ZF598 and ZF552)^26^ and demonstrated that both proteins catalyzed the incorporation of DNAme and induced potent cell growth arrest. This effect was dependent on both a functional DBD and the catalytic function of DNMT3A, as an untargeted DNMT3A construct or the expression of a catalytic mutant resulted in an unspecific background incorporation of DNAme, which was not capable of silencing *SOX2* or inhibit cell growth. Furthermore, *de novo* DNAme was stably maintained *in vivo* even 53 days after removal of the ZF598-DNMT3A and was accompanied with a sustained suppression of *SOX2* expression and tumor growth inhibition. It is important to note that the absence of expression of the methyltransferase upon long-term removal of Dox was carefully validated using immunoblotting and immunofluorescence.

In this study, we utilized modular ZF proteins engineered to bind an 18-bp sequence in the core promoter of *SOX2*. ZFs are well-characterized DBDs and have been used for almost two decades for DNA targeting with customizable sequence selectivity.^27^ Furthermore, recent novel approaches to target endogenous gene expression also hold great promise such as transcription activator-like effectors^28, 29^ and the RNA-guided clustered regularly interspersed short palindromic repeats (Cas) system.^30, 31^ Both transcription activator-like effectors and Cas9 could be similarly used as alternatives to ZFs to target the catalytic active domain of DNMT3A to specific chromosomal sites.

Pioneering work has been recently published by Konermann *et al.*^32^ who fused 32 different histone effector domains to a transcription activator-like effector DBD targeting the *Neurog2* locus and demonstrated transcriptional repression. However, the spatio-temporal dynamics associated with *de novo* DNAme and histone post-transcriptional modifications still remain elusive. Here we demonstrate that *de novo* DNAme is faithfully transmitted after clearance of expression of the 6ZF-DNMT3A fusions. The artificial incorporation of DNAme provides an initial platform, which is read, written and propagated by endogenous methylation machinery during DNA replication. The complex between DNMT1, UHRF1 (ubiquitin-like PHD (containing plant homeodomain) and RING (really interesting new gene) finger domains 1) and PCNA (proliferating cell nuclear antigen) at the replication fork could mediate the cross-talk between methylated DNA and repressive histone modifications.^33, 34^ In our experiments, it is conceivable that such mechanisms could be responsible for the maintenance of DNAme and the downregulation of *SOX2*.^35^

In addition to hereditable transmission during mitosis, we observed that the *de novo* methylation in the *SOX2* locus was expanded at least 1 Kbp away from the targeted site. Potential mechanisms could involve the endogenous spreading of DNAme for example by chromatin looping or iterative reading and writing of DNA and histone post-transcriptional modifications,^36^ or by low-frequency occupancy of these sites by the 6ZF domains. In this regard, it will be interesting to investigate whether this effect is also observed with more recently developed engineering approaches such as transcription activator-like effectors or clustered regularly interspersed short palindromic repeats/dCas9.

In contrast with DNMT3A, cells expressing the ZF-SKD fusions did not sustain a stable downregulation of *SOX2*, indicating mechanistically distinct epigenetic processes initiated by the ZF-DNMT3A and ZF-SKD constructs. SKD mediates silencing of target genes through recruitment of KAP1 (Krueppel-associated protein 1), which acts as a scaffold for heterochromatin-inducing modifiers such as HP1 and methyltransferase SETDB1.^36^ However, unlike DNMT3A, SKD has no intrinsic enzymatic activity, and mainly carries a recruiting activity. In our study, the suppression of SKD expression did not result in epigenetic memory.

To date, four epigenetic drugs have been approved by the US Food and Drug Administration, including two DNMT and two HDAC inhibitors^37^ to reactivate aberrantly silenced tumor suppressor genes. Here we report a novel strategy that will enable the silencing of aberrantly expressed oncogenes. We have previously demonstrated systemic delivery of lipid-protamine-RNA nanoparticles encapsulating mRNA encoding a ZF protein upregulating the *MASPIN* promoter for the treatment of serous ovarian cancer.^38^ Such lipid-protamine-RNA technology could be similarly adapted to deliver the ZF-DNMT3A constructs for stable heterochromatization of *SOX2*. The small, compact molecular architecture of ZF domains and their lack of immunogenicity make them very suitable molecular scaffolds for this type of delivery.

In summary, we demonstrate the applicability of DBD-DNMT3A fusions to induce targeted DNAme to stably repress oncogenic expression in a long-term xenograft mouse model of breast cancer. This approach could be extended to many other oncogenic drivers for which no drug is currently available to promote potent and durable cancer cell growth inhibition. In addition to its important implications in cancer therapeutics, our approach provides a platform to induce targeted DNAme and investigate the temporal and spatial propagation of this epigenetic state and its effects on gene expression on a genomic level *in vivo*.

## Materials and methods

### Generation of stable cell lines

The construction of the SKD, DNMT3A, DNMT3A-E74A and the 6ZF domains has been described elsewhere.^17, 19^ ZF598-DNMT3A and ZF598-DNMT3A-E74A were cloned into pRetroX-Tight-Pur (CloneTech, Mountain View, CA, USA). Generation of MCF7 cells stably expressing ZF598-DNMT3A and ZF598-DNMT3A-E74A was performed as described.^17^ Cells were induced with Dox every 48 h and either harvested at 72 h after the first induction (+Dox) or removed from Dox and subcultured for 8 days and processed for western blot.

### Cell proliferation assays

Eighteen replicates of MCF7 cells stably expressing empty vector, ZF598-SKD and ZF598-DNMT3A were plated in 96-wells plates (1000 cells/well). Twelve replicates were induced with Dox at time point 0 and after 48 h. After 72 h, six replicates were removed from Dox, whereas six replicates were continuously induced. Cell proliferation was assessed by CellTiterGlo assay (Promega, Madison, WI, USA) every 24 h for a total period of 144 h.^39^ Results were normalized to readings at day 0.

### Bisulfite conversion and MassARRAY methylation analysis

After genomic DNA extraction, 2 μg of sample DNA (derived from either cell line or tumor) was treated with sodium bisulfite using the EZ DNA Methylation-Direct Kit (Zymo Research, Irvine, CA, USA). We custom designed primers for three amplicons spanning the core *SOX2* promoter, one specifically including the 6ZF-binding site 5′-gGCCCCCTCCTCCCCCGGC-3′ and two amplicons up- and downstream of the 6ZF-binding site. Polymerase chain reaction was then carried out on 5–10 ng of sodium bisulfite converted sample DNA using conversion-specific primers. For amplicon I: forward primer 5′-aggaagagagGGATAGAGGTTTGGGTTTTTTAATTT-3′ and reverse primer 5′-cagtaatacgactcactatagggagaaggctAAACCAACCTACCAACCACTAAAA-3′. For amplicon II: forward primer 5′-aggaagagagAAAGGTTTTTTAGTGGTTGGTAGGT-3′ and 5′-agtaatacgactcactatagggagaaggctAAAACTCAAACTTCTCTCCCTTTCT-3′ reverse primer. For amplicon III: 5′-aggaagagagTTTTGGTATGGTTTTTGGTTTTATG-3′ forward primer and 5′-cagtaatacgactcactatagggagaaggctAATTTTCTCCATACTATTTCTTACTCTCC-3′ reverse primer with lower case letters representing the T7 tag sequences. Percent *SOX2* DNAme was quantified using mass spectrometry with the SEQUENOM EpiTYPER T complete reagent kit (San Diego, CA, USA). Conventional sodium bisulfite-sequencing analysis was carried out as described before using 5′-AAAGGTTTTTTAGTGGTTGGTAGGT-3′ forward primer and 5′-AAAACTCAAACTTCTCTCCCTTTCT-3′ reverse primer for polymerase chain reaction amplification of bisulfite converted DNA.^19^ Primers to detect the % methylation for the MASPIN amplicon using the EpiTYPER platform: forward primer 5′-aggaagagagGAGGTTTTTTGGAAGTTGTGTAGAT-3′ and reverse primer 5′-cagtaatacgactcactatagggagaaggctCCCCACCTTACTTACCTAAAATCAC-3′

### Mouse experiments

Female NUDE mice (age 4 weeks) were purchased from Taconic Farms (Hudson, NY, USA) and housed under pathogen-free conditions. The Institutional Animal Care and Use Committee at the University of North Carolina at Chapel Hill approved all experiments described herein. Estrogen pellets containing 2 mg 17β-Estradiol (Sigma-Aldrich Corp., St Louis, MO, USA) and 8 mg Cellulose (Sigma-Aldrich Corp.) were subcutaneously implanted in the animals 7 days prior of the injection of the cells. MCF7 cells (2 × 10^6^) were collected and resuspended with matrigel (BD Bioscience, San Diego, CA, USA) 1:1 volume ratio in a total volume of 100 ml. The cell–matrigel mixture was injected into the mouse flank of *N*=11 mice for empty vector and *N*=22 for ZF598-DNMT3A. Tumor growth was monitored by caliper twice a week. When the tumor reached a size of ~25–30 mm^3^, Dox was administered to the mice in the form of green food pellets (200 mg/kg of mice chow) for a period of 19 days. At day 19 post induction half of the animals of each group were removed from Dox, whereas the other half was maintained under Dox diet. During the entire experiment, the mice weight was monitored to ensure absence of toxicity. Animals were killed when tumor reached 100 mm^3^ (empty vector 29 days post induction, ZF598-DNMT3A upon Dox removal (‘No Dox') for 43 days post induction and ZF598-DNMT3A Dox removal at 72 days post induction). Statistical differences between control and ZF-DNMT3A animals were assessed by Wilcoxon Ranks Sum Test analysis.

## References

[bib1] 1Kouzarides T. Chromatin modifications and their function. Cell 2007; 128: 693–705. 17320507 10.1016/j.cell.2007.02.005

[bib2] 2Beisel C, Paro R. Silencing chromatin: comparing modes and mechanisms. Nat Rev Genet 2011; 12: 123–135. 21221116 10.1038/nrg2932

[bib3] 3Lange UC, Schneider R. What an epigenome remembers. Bioessays 2010; 32: 659–668. 20658704 10.1002/bies.201000030

[bib4] 4Sandoval J, Esteller M. Cancer epigenomics: beyond genomics. Curr Opin Genet Dev 2012; 22: 50–55. 22402447 10.1016/j.gde.2012.02.008

[bib5] 5The Cancer Genome Atlas Network. Comprehensive molecular portraits of human breast tumours. Nature 2012; 490: 61–70. 23000897 10.1038/nature11412 PMC3465532

[bib6] 6Stephens PJ, Tarpey PS, Davies H, Van Loo P, Greenman C, Wedge DC et al. The landscape of cancer genes and mutational processes in breast cancer. Nature 2012; 486: 400–404. 22722201 10.1038/nature11017 PMC3428862

[bib7] 7Curtis C, Shah SP, Chin SF, Turashvili G, Rueda OM, Dunning MJ et al. The genomic and transcriptomic architecture of 2,000 breast tumours reveals novel subgroups. Nature 2012; 486: 346–352. 22522925 10.1038/nature10983 PMC3440846

[bib8] 8Boyer LA, Lee TI, Cole MF, Johnstone SE, Levine SS, Zucker JP et al. Core transcriptional regulatory circuitry in human embryonic stem cells. Cell 2005; 122: 947–956. 16153702 10.1016/j.cell.2005.08.020 PMC3006442

[bib9] 9Brazel CY, Limke TL, Osborne JK, Miura T, Cai J, Pevny L et al. Sox2 expression defines a heterogeneous population of neurosphere-forming cells in the adult murine brain. Aging Cell 2005; 4: 197–207. 16026334 10.1111/j.1474-9726.2005.00158.x

[bib10] 10Sikorska M, Sandhu JK, Deb-Rinker P, Jezierski A, Leblanc J, Charlebois C et al. Epigenetic modifications of SOX2 enhancers, SRR1 and SRR2, correlate with *in vitro* neural differentiation. J Neurosci Res 2008; 86: 1680–1693. 18293417 10.1002/jnr.21635

[bib11] 11Sussman RT, Stanek TJ, Esteso P, Gearhart JD, Knudsen KE, McMahon SB. The epigenetic modifier ubiquitin-specific protease 22 (USP22) regulates embryonic stem cell differentiation via transcriptional repression of sex-determining region Y-box 2 (SOX2). J Biol Chem 2013; 288: 24234–24246. 23760504 10.1074/jbc.M113.469783 PMC3745368

[bib12] 12Schmitz M, Temme A, Senner V, Ebner R, Schwind S, Stevanovic S et al. Identification of SOX2 as a novel glioma-associated antigen and potential target for T cell-based immunotherapy. Br J Cancer 2007; 96: 1293–1301. 17375044 10.1038/sj.bjc.6603696 PMC2360145

[bib13] 13Bareiss PM, Paczulla A, Wang H, Schairer R, Wiehr S, Kohlhofer U et al. SOX2 expression associates with stem cell state in human ovarian carcinoma. Cancer Res 2013; 73: 5544–5555. 23867475 10.1158/0008-5472.CAN-12-4177

[bib14] 14Bass AJ, Watanabe H, Mermel CH, Yu S, Perner S, Verhaak RG et al. SOX2 is an amplified lineage-survival oncogene in lung and esophageal squamous cell carcinomas. Nat Genet 2009; 41: 1238–1242. 19801978 10.1038/ng.465 PMC2783775

[bib15] 15Alonso MM, Diez-Valle R, Manterola L, Rubio A, Liu D, Cortes-Santiago N et al. Genetic and epigenetic modifications of Sox2 contribute to the invasive phenotype of malignant gliomas. PLoS ONE 2011; 6: e26740. 22069467 10.1371/journal.pone.0026740 PMC3206066

[bib16] 16Chen Y, Shi L, Zhang L, Li R, Liang J, Yu W et al. The molecular mechanism governing the oncogenic potential of SOX2 in breast cancer. J Biol Chem 2008; 283: 17969–17978. 18456656 10.1074/jbc.M802917200

[bib17] 17Stolzenburg S, Rots MG, Beltran AS, Rivenbark AG, Yuan X, Qian H et al. Targeted silencing of the oncogenic transcription factor SOX2 in breast cancer. Nucleic Acids Res 2012; 40: 6725–6740. 22561374 10.1093/nar/gks360 PMC3413152

[bib18] 18Xiang R, Liao D, Cheng T, Zhou H, Shi Q, Chuang TS et al. Downregulation of transcription factor SOX2 in cancer stem cells suppresses growth and metastasis of lung cancer. Br J Cancer 2011; 104: 1410–1417. 21468047 10.1038/bjc.2011.94 PMC3101944

[bib19] 19Rivenbark AG, Stolzenburg S, Beltran AS, Yuan X, Rots MG, Strahl BD et al. Epigenetic reprogramming of cancer cells via targeted DNA methylation. Epigenetics 2012; 7: 350–360. 22419067 10.4161/epi.19507 PMC3368819

[bib20] 20Siddique AN, Nunna S, Rajavelu A, Zhang Y, Jurkowska RZ, Reinhardt R et al. Targeted methylation and gene silencing of VEGF-A in human cells by using a designed Dnmt3a-Dnmt3L single-chain fusion protein with increased DNA methylation activity. J Mol Biol 2013; 425: 479–491. 23220192 10.1016/j.jmb.2012.11.038

[bib21] 21Li F, Papworth M, Minczuk M, Rohde C, Zhang Y, Ragozin S et al. Chimeric DNA methyltransferases target DNA methylation to specific DNA sequences and repress expression of target genes. Nucleic Acids Res 2007; 35: 100–112. 17151075 10.1093/nar/gkl1035 PMC1761428

[bib22] 22de Groote ML, Verschure PJ, Rots MG. Epigenetic Editing: targeted rewriting of epigenetic marks to modulate expression of selected target genes. Nucleic Acids Res 2012; 40: 10596–10613. 23002135 10.1093/nar/gks863 PMC3510492

[bib23] 23Nunna S, Reinhardt R, Ragozin S, Jeltsch A. Targeted methylation of the epithelial cell adhesion molecule (EpCAM) promoter to silence its expression in ovarian cancer cells. PLoS ONE 2014; 9: e87703. 24489952 10.1371/journal.pone.0087703 PMC3906225

[bib24] 24Chaikind B, Ostermeier M. Directed evolution of improved zinc finger methyltransferases. PLoS ONE 2014; 9: e96931. 24810747 10.1371/journal.pone.0096931 PMC4014571

[bib25] 25Blancafort P, Jin J, Frye S. Writing and rewriting the epigenetic code of cancer cells: from engineered proteins to small molecules. Mol Pharmacol 2013; 83: 563–576. 23150486 10.1124/mol.112.080697 PMC3920093

[bib26] 26Stolzenburg S, Bilsland A, Keith WN, Rots MG. Modulation of gene expression using zinc finger-based artificial transcription factors. Methods Mol Biol 2010; 649: 117–132. 20680831 10.1007/978-1-60761-753-2_7

[bib27] 27Choo Y, Sanchez-Garcia I, Klug A. *In vivo* repression by a site-specific DNA-binding protein designed against an oncogenic sequence. Nature 1994; 372: 642–645. 7990954 10.1038/372642a0

[bib28] 28Boch J, Scholze H, Schornack S, Landgraf A, Hahn S, Kay S et al. Breaking the code of DNA binding specificity of TAL-type III effectors. Science 2009; 326: 1509–1512. 19933107 10.1126/science.1178811

[bib29] 29Bogdanove AJ, Voytas DF. TAL effectors: customizable proteins for DNA targeting. Science 2011; 333: 1843–1846. 21960622 10.1126/science.1204094

[bib30] 30Jinek M, Chylinski K, Fonfara I, Hauer M, Doudna JA, Charpentier E. A programmable dual-RNA-guided DNA endonuclease in adaptive bacterial immunity. Science 2012; 337: 816–821. 22745249 10.1126/science.1225829 PMC6286148

[bib31] 31Wiedenheft B, Sternberg SH, Doudna JA. RNA-guided genetic silencing systems in bacteria and archaea. Nature 2012; 482: 331–338. 22337052 10.1038/nature10886

[bib32] 32Konermann S, Brigham MD, Trevino A, Hsu PD, Heidenreich M, Le C et al. Optical control of mammalian endogenous transcription and epigenetic states. Nature 2013. 10.1038/nature12466 PMC3856241 23877069

[bib33] 33Fuks F, Burgers WA, Brehm A, Hughes-Davies L, Kouzarides T. DNA methyltransferase Dnmt1 associates with histone deacetylase activity. Nat Gen 2000; 24: 88–91. 10.1038/71750 10615135

[bib34] 34Esteve PO, Chin HG, Smallwood A, Feehery GR, Gangisetty O, Karpf AR et al. Direct interaction between DNMT1 and G9a coordinates DNA and histone methylation during replication. Genes Dev 2006; 20: 3089–3103. 17085482 10.1101/gad.1463706 PMC1635145

[bib35] 35Rodriguez J, Munoz M, Vives L, Frangou CG, Groudine M, Peinado MA. Bivalent domains enforce transcriptional memory of DNA methylated genes in cancer cells. Proc Natl Acad Sci USA 2008; 105: 19809–19814. 19060200 10.1073/pnas.0810133105 PMC2596747

[bib36] 36Hathaway NA, Bell O, Hodges C, Miller EL, Neel DS, Crabtree GR. Dynamics and memory of heterochromatin in living cells. Cell 2012; 149: 1447–1460. 22704655 10.1016/j.cell.2012.03.052 PMC3422694

[bib37] 37Heyn H, Esteller M. DNA methylation profiling in the clinic: applications and challenges. Nat Rev Genet 2012; 13: 679–692. 22945394 10.1038/nrg3270

[bib38] 38Lara H, Wang Y, Beltran AS, Juarez-Moreno K, Yuan X, Kato S et al. Targeting serous epithelial ovarian cancer with designer zinc finger transcription factors. J Biol Chem 2012; 287: 29873–29886. 22782891 10.1074/jbc.M112.360768 PMC3436144

[bib39] 39Beltran AS, Rivenbark AG, Richardson BT, Yuan X, Quian H, Hunt JP et al. Generation of tumor initiating cells by exogenous delivery of OCT4 Transcription Factor. Breast Cancer Res 2011; 13: R94. 21952072 10.1186/bcr3019 PMC3262206

