# Supplementary material for: Stable oncogenic silencing in vivo by programmable and targeted de novo DNA methylation in breast cancer
Source: Oncogene. 2015 Feb 16;34(43):5427–35. doi: 10.1038/onc.2014.470 (PMC4633433; doi:10.1038/onc.2014.470)
Supplement: Supplementary Table S1 [file onc2014470x1.doc]

**Table S1:** Percent DNA methylation in the *SOX2* Amplicon I (annotated as SOX2_002_446bp). Mean of methylation values for CpG dinucleotides were obtained using the EpiTYPER MassARRAY platform with genomic DNA derived from the tumor xenografts sampled from mice with the following five treatments below. The same letter (e.g., 438 and 580 are both "A and A") indicates that these means were not significantly different from each other (See also supplemental Figure S4). Conversely, a different letter means each t-test comparison was significantly different at <0.05. ANOVA p-values for all treatments for each CpG unit are provided. MassARRAY experiments were independently performed at least two times.

**TUMOR SAMPLES Treatment Description**

1_438 ZF598-DNMT3A +Dox 43 days post-induction

2_580 ZF598-DNMT3A Dox removal 43 days post-induction

3_435 Empty vector + Dox 29 days post-induction*

4_583 Empty vector -Dox 29 days post-induction*

5_444 ZF598-DNMT3A -Dox 43 days post-induction

**Control animals were sacrificed at day 29 post-induction due to the large size of the tumor.*

| **Amplicon I (SOX2_002) by CpG unit** | **Tumor ID** | **Group A** | **Group B** | **Group C** | **Fraction Methylation** |
| --- | --- | --- | --- | --- | --- |
| CpG_1 | 2_580 | A |  |  | 0.105 |
| CpG_1 | 1_438 | A |  |  | 0.090 |
| CpG_1 | 4_583 |  | B |  | 0.035 |
| CpG_1 | 3_435 |  | B | C | 0.020 |
| CpG_1 | 5_444 |  |  | C | 0.010 |
| CpG_2 | 2_580 | A |  |  | 0.340 |
| CpG_2 | 1_438 | A |  |  | 0.325 |
| CpG_2 | 3_435 |  | B |  | 0.080 |
| CpG_2 | 4_583 |  | B |  | 0.070 |
| CpG_2 | 5_444 |  |  | C | 0.030 |
| CpG_3 | 1_438 | A |  |  | 0.460 |
| CpG_3 | 4_583 |  | B |  | 0.220 |
| CpG_3 | 3_435 |  | B |  | 0.190 |
| CpG_3 | 2_580 |  | B |  | 0.180 |
| CpG_3 | 5_444 |  | B |  | 0.160 |
| CpG_4 | 1_438 | A |  |  | 0.395 |
| CpG_4 | 2_580 | A | B |  | 0.240 |
| CpG_4 | 5_444 |  | B |  | 0.160 |
| CpG_4 | 4_583 |  | B |  | 0.110 |
| CpG_4 | 3_435 |  | B |  | 0.075 |
| CpG_5 | 1_438 | A |  |  | 0.460 |
| CpG_5 | 4_583 |  | B |  | 0.220 |
| CpG_5 | 3_435 |  | B |  | 0.190 |
| CpG_5 | 2_580 |  | B |  | 0.180 |
| CpG_5 | 5_444 |  | B |  | 0.160 |
| CpG_6 | 2_580 | A |  |  | 0.195 |
| CpG_6 | 1_438 |  | B |  | 0.160 |
| CpG_6 | 3_435 |  |  | C | 0.020 |
| CpG_6 | 4_583 |  |  | C | 0.000 |
| CpG_6 | 5_444 |  |  | C | 0.000 |
| CpG_7 | 2_580 | A |  |  | 0.210 |
| CpG_7 | 1_438 |  | B |  | 0.120 |
| CpG_7 | 4_583 |  |  | C | 0.010 |
| CpG_7 | 3_435 |  |  | C | 0.005 |
| CpG_7 | 5_444 |  |  | C | 0.000 |
| CpG_9 | 2_580 | A |  |  | 0.120 |
| CpG_9 | 3_435 |  | B |  | 0.040 |
| CpG_9 | 5_444 |  | B |  | 0.040 |
| CpG_9 | 1_438 |  | B |  | 0.035 |
| CpG_9 | 4_583 |  | B |  | 0.020 |
| CpG_10 | 2_580 | A |  |  | 0.350 |
| CpG_10 | 1_438 |  | B |  | 0.225 |
| CpG_10 | 3_435 |  |  | C | 0.060 |
| CpG_10 | 4_583 |  |  | C | 0.055 |
| CpG_10 | 5_444 |  |  | C | 0.055 |
| CpG_11.12 | 2_580 | A |  |  | 0.215 |
| CpG_11.12 | 1_438 | A |  |  | 0.165 |
| CpG_11.12 | 3_435 |  | B |  | 0.070 |
| CpG_11.12 | 4_583 |  | B |  | 0.060 |
| CpG_11.12 | 5_444 |  | B |  | 0.060 |
| CpG_14 | 2_580 | A |  |  | 0.065 |
| CpG_14 | 3_435 | A |  |  | 0.050 |
| CpG_14 | 4_583 | A |  |  | 0.045 |
| CpG_14 | 1_438 | A |  |  | 0.015 |
| CpG_14 | 5_444 | A |  |  | 0.005 |
| CpG_15 | 2_580 | A |  |  | 0.125 |
| CpG_15 | 1_438 | A |  |  | 0.085 |
| CpG_15 | 4_583 | A |  |  | 0.075 |
| CpG_15 | 5_444 | A |  |  | 0.075 |
| CpG_15 | 3_435 | A |  |  | 0.070 |
| CpG_16 | 2_580 | A |  |  | 0.070 |
| CpG_16 | 1_438 |  | B |  | 0.025 |
| CpG_16 | 4_583 |  | B |  | 0.025 |
| CpG_16 | 3_435 |  |  | C | 0.010 |
| CpG_16 | 5_444 |  |  | C | 0.010 |
| Average | 1_438 | A |  |  | 0.211 |
| Average | 2_580 | A |  |  | 0.202 |
| Average | 5_444 |  | B |  | 0.080 |
| Average | 4_583 |  | B |  | 0.077 |
| Average | 3_435 |  | B |  | 0.073 |

| **Amplicon I (SOX2_002)**  **by CpG unit** | **Compare 1** | **Compare 2** | **Difference** | **Std Err Diff** | **Lower CL** | **Upper CL** | **p-value t-test** |
| --- | --- | --- | --- | --- | --- | --- | --- |
| CpG_1 | 2_580 | 5_444 | 0.095 | 0.0077 | 0.0751 | 0.1149 | <.0001 |
| CpG_1 | 2_580 | 3_435 | 0.085 | 0.0077 | 0.0651 | 0.1049 | 0.0001 |
| CpG_1 | 1_438 | 5_444 | 0.080 | 0.0077 | 0.0601 | 0.0999 | 0.0001 |
| CpG_1 | 2_580 | 4_583 | 0.070 | 0.0077 | 0.0501 | 0.0899 | 0.0003 |
| CpG_1 | 1_438 | 3_435 | 0.070 | 0.0077 | 0.0501 | 0.0899 | 0.0003 |
| CpG_1 | 1_438 | 4_583 | 0.055 | 0.0077 | 0.0351 | 0.0749 | 0.0009 |
| CpG_1 | 4_583 | 5_444 | 0.025 | 0.0077 | 0.0051 | 0.0449 | 0.0233 |
| CpG_1 | 2_580 | 1_438 | 0.015 | 0.0077 | -0.0049 | 0.0349 | 0.1106 |
| CpG_1 | 4_583 | 3_435 | 0.015 | 0.0077 | -0.0049 | 0.0349 | 0.1106 |
| CpG_1 | 3_435 | 5_444 | 0.010 | 0.0077 | -0.0099 | 0.0299 | 0.2532 |
| CpG_2 | 2_580 | 5_444 | 0.310 | 0.0114 | 0.2807 | 0.3393 | <.0001 |
| CpG_2 | 1_438 | 5_444 | 0.295 | 0.0114 | 0.2657 | 0.3243 | <.0001 |
| CpG_2 | 2_580 | 4_583 | 0.270 | 0.0114 | 0.2407 | 0.2993 | <.0001 |
| CpG_2 | 2_580 | 3_435 | 0.260 | 0.0114 | 0.2307 | 0.2893 | <.0001 |
| CpG_2 | 1_438 | 4_583 | 0.255 | 0.0114 | 0.2257 | 0.2843 | <.0001 |
| CpG_2 | 1_438 | 3_435 | 0.245 | 0.0114 | 0.2157 | 0.2743 | <.0001 |
| CpG_2 | 3_435 | 5_444 | 0.050 | 0.0114 | 0.0207 | 0.0793 | 0.0071 |
| CpG_2 | 4_583 | 5_444 | 0.040 | 0.0114 | 0.0107 | 0.0693 | 0.0171 |
| CpG_2 | 2_580 | 1_438 | 0.015 | 0.0114 | -0.0143 | 0.0443 | 0.2454 |
| CpG_2 | 3_435 | 4_583 | 0.010 | 0.0114 | -0.0193 | 0.0393 | 0.4206 |
| CpG_3 | 1_438 | 5_444 | 0.300 | 0.0743 | 0.1090 | 0.4910 | 0.0099 |
| CpG_3 | 1_438 | 2_580 | 0.280 | 0.0743 | 0.0890 | 0.4710 | 0.0130 |
| CpG_3 | 1_438 | 3_435 | 0.270 | 0.0743 | 0.0790 | 0.4610 | 0.0150 |
| CpG_3 | 1_438 | 4_583 | 0.240 | 0.0743 | 0.0490 | 0.4310 | 0.0232 |
| CpG_3 | 4_583 | 5_444 | 0.060 | 0.0743 | -0.1310 | 0.2510 | 0.4560 |
| CpG_3 | 4_583 | 2_580 | 0.040 | 0.0743 | -0.1510 | 0.2310 | 0.6134 |
| CpG_3 | 4_583 | 3_435 | 0.030 | 0.0743 | -0.1610 | 0.2210 | 0.7031 |
| CpG_3 | 3_435 | 5_444 | 0.030 | 0.0743 | -0.1610 | 0.2210 | 0.7031 |
| CpG_3 | 2_580 | 5_444 | 0.020 | 0.0743 | -0.1710 | 0.2110 | 0.7985 |
| CpG_3 | 3_435 | 2_580 | 0.010 | 0.0743 | -0.1810 | 0.2010 | 0.8982 |
| CpG_4 | 1_438 | 3_435 | 0.320 | 0.0898 | 0.0892 | 0.5508 | 0.0161 |
| CpG_4 | 1_438 | 4_583 | 0.285 | 0.0898 | 0.0542 | 0.5158 | 0.0247 |
| CpG_4 | 1_438 | 5_444 | 0.235 | 0.0898 | 0.0042 | 0.4658 | 0.0472 |
| CpG_4 | 2_580 | 3_435 | 0.165 | 0.0898 | -0.0658 | 0.3958 | 0.1255 |
| CpG_4 | 1_438 | 2_580 | 0.155 | 0.0898 | -0.0758 | 0.3858 | 0.1448 |
| CpG_4 | 2_580 | 4_583 | 0.130 | 0.0898 | -0.1008 | 0.3608 | 0.2073 |
| CpG_4 | 5_444 | 3_435 | 0.085 | 0.0898 | -0.1458 | 0.3158 | 0.3872 |
| CpG_4 | 2_580 | 5_444 | 0.080 | 0.0898 | -0.1508 | 0.3108 | 0.4137 |
| CpG_4 | 5_444 | 4_583 | 0.050 | 0.0898 | -0.1808 | 0.2808 | 0.6016 |
| CpG_4 | 4_583 | 3_435 | 0.035 | 0.0898 | -0.1958 | 0.2658 | 0.7127 |
| CpG_5 | 1_438 | 5_444 | 0.300 | 0.0743 | 0.1090 | 0.4910 | 0.0099 |
| CpG_5 | 1_438 | 2_580 | 0.280 | 0.0743 | 0.0890 | 0.4710 | 0.0130 |
| CpG_5 | 1_438 | 3_435 | 0.270 | 0.0743 | 0.0790 | 0.4610 | 0.0150 |
| CpG_5 | 1_438 | 4_583 | 0.240 | 0.0743 | 0.0490 | 0.4310 | 0.0232 |
| CpG_5 | 4_583 | 5_444 | 0.060 | 0.0743 | -0.1310 | 0.2510 | 0.4560 |
| CpG_5 | 4_583 | 2_580 | 0.040 | 0.0743 | -0.1510 | 0.2310 | 0.6134 |
| CpG_5 | 4_583 | 3_435 | 0.030 | 0.0743 | -0.1610 | 0.2210 | 0.7031 |
| CpG_5 | 3_435 | 5_444 | 0.030 | 0.0743 | -0.1610 | 0.2210 | 0.7031 |
| CpG_5 | 2_580 | 5_444 | 0.020 | 0.0743 | -0.1710 | 0.2110 | 0.7985 |
| CpG_5 | 3_435 | 2_580 | 0.010 | 0.0743 | -0.1810 | 0.2010 | 0.8982 |
| CpG_6 | 2_580 | 4_583 | 0.195 | 0.0095 | 0.1706 | 0.2194 | <.0001 |
| CpG_6 | 2_580 | 5_444 | 0.195 | 0.0095 | 0.1706 | 0.2194 | <.0001 |
| CpG_6 | 2_580 | 3_435 | 0.175 | 0.0095 | 0.1506 | 0.1994 | <.0001 |
| CpG_6 | 1_438 | 4_583 | 0.160 | 0.0095 | 0.1356 | 0.1844 | <.0001 |
| CpG_6 | 1_438 | 5_444 | 0.160 | 0.0095 | 0.1356 | 0.1844 | <.0001 |
| CpG_6 | 1_438 | 3_435 | 0.140 | 0.0095 | 0.1156 | 0.1644 | <.0001 |
| CpG_6 | 2_580 | 1_438 | 0.035 | 0.0095 | 0.0106 | 0.0594 | 0.0142 |
| CpG_6 | 3_435 | 4_583 | 0.020 | 0.0095 | -0.0044 | 0.0444 | 0.0888 |
| CpG_6 | 3_435 | 5_444 | 0.020 | 0.0095 | -0.0044 | 0.0444 | 0.0888 |
| CpG_6 | 5_444 | 4_583 | 0.000 | 0.0095 | -0.0244 | 0.0244 | 1.0000 |
| CpG_7 | 2_580 | 5_444 | 0.210 | 0.0158 | 0.1694 | 0.2506 | <.0001 |
| CpG_7 | 2_580 | 3_435 | 0.205 | 0.0158 | 0.1644 | 0.2456 | <.0001 |
| CpG_7 | 2_580 | 4_583 | 0.200 | 0.0158 | 0.1594 | 0.2406 | <.0001 |
| CpG_7 | 1_438 | 5_444 | 0.120 | 0.0158 | 0.0794 | 0.1606 | 0.0006 |
| CpG_7 | 1_438 | 3_435 | 0.115 | 0.0158 | 0.0744 | 0.1556 | 0.0008 |
| CpG_7 | 1_438 | 4_583 | 0.110 | 0.0158 | 0.0694 | 0.1506 | 0.0009 |
| CpG_7 | 2_580 | 1_438 | 0.090 | 0.0158 | 0.0494 | 0.1306 | 0.0023 |
| CpG_7 | 4_583 | 5_444 | 0.010 | 0.0158 | -0.0306 | 0.0506 | 0.5549 |
| CpG_7 | 4_583 | 3_435 | 0.005 | 0.0158 | -0.0356 | 0.0456 | 0.7646 |
| CpG_7 | 3_435 | 5_444 | 0.005 | 0.0158 | -0.0356 | 0.0456 | 0.7646 |
| CpG_9 | 2_580 | 4_583 | 0.100 | 0.0182 | 0.0533 | 0.1467 | 0.0027 |
| CpG_9 | 2_580 | 1_438 | 0.085 | 0.0182 | 0.0383 | 0.1317 | 0.0054 |
| CpG_9 | 2_580 | 3_435 | 0.080 | 0.0182 | 0.0333 | 0.1267 | 0.0070 |
| CpG_9 | 2_580 | 5_444 | 0.080 | 0.0182 | 0.0333 | 0.1267 | 0.0070 |
| CpG_9 | 3_435 | 4_583 | 0.020 | 0.0182 | -0.0267 | 0.0667 | 0.3211 |
| CpG_9 | 5_444 | 4_583 | 0.020 | 0.0182 | -0.0267 | 0.0667 | 0.3211 |
| CpG_9 | 1_438 | 4_583 | 0.015 | 0.0182 | -0.0317 | 0.0617 | 0.4466 |
| CpG_9 | 3_435 | 1_438 | 0.005 | 0.0182 | -0.0417 | 0.0517 | 0.7941 |
| CpG_9 | 5_444 | 1_438 | 0.005 | 0.0182 | -0.0417 | 0.0517 | 0.7941 |
| CpG_9 | 5_444 | 3_435 | 0.000 | 0.0182 | -0.0467 | 0.0467 | 1.0000 |
| CpG_10 | 2_580 | 4_583 | 0.295 | 0.0138 | 0.2596 | 0.3304 | <.0001 |
| CpG_10 | 2_580 | 5_444 | 0.295 | 0.0138 | 0.2596 | 0.3304 | <.0001 |
| CpG_10 | 2_580 | 3_435 | 0.290 | 0.0138 | 0.2546 | 0.3254 | <.0001 |
| CpG_10 | 1_438 | 4_583 | 0.170 | 0.0138 | 0.1346 | 0.2054 | <.0001 |
| CpG_10 | 1_438 | 5_444 | 0.170 | 0.0138 | 0.1346 | 0.2054 | <.0001 |
| CpG_10 | 1_438 | 3_435 | 0.165 | 0.0138 | 0.1296 | 0.2004 | <.0001 |
| CpG_10 | 2_580 | 1_438 | 0.125 | 0.0138 | 0.0896 | 0.1604 | 0.0003 |
| CpG_10 | 3_435 | 4_583 | 0.005 | 0.0138 | -0.0304 | 0.0404 | 0.7316 |
| CpG_10 | 3_435 | 5_444 | 0.005 | 0.0138 | -0.0304 | 0.0404 | 0.7316 |
| CpG_10 | 5_444 | 4_583 | 0.000 | 0.0138 | -0.0354 | 0.0354 | 1.0000 |
| CpG_11.12 | 2_580 | 4_583 | 0.155 | 0.0272 | 0.0851 | 0.2249 | 0.0023 |
| CpG_11.12 | 2_580 | 5_444 | 0.155 | 0.0272 | 0.0851 | 0.2249 | 0.0023 |
| CpG_11.12 | 2_580 | 3_435 | 0.145 | 0.0272 | 0.0751 | 0.2149 | 0.0031 |
| CpG_11.12 | 1_438 | 4_583 | 0.105 | 0.0272 | 0.0351 | 0.1749 | 0.0119 |
| CpG_11.12 | 1_438 | 5_444 | 0.105 | 0.0272 | 0.0351 | 0.1749 | 0.0119 |
| CpG_11.12 | 1_438 | 3_435 | 0.095 | 0.0272 | 0.0251 | 0.1649 | 0.0174 |
| CpG_11.12 | 2_580 | 1_438 | 0.050 | 0.0272 | -0.0199 | 0.1199 | 0.1255 |
| CpG_11.12 | 3_435 | 5_444 | 0.010 | 0.0272 | -0.0599 | 0.0799 | 0.7282 |
| CpG_11.12 | 3_435 | 4_583 | 0.010 | 0.0272 | -0.0599 | 0.0799 | 0.7282 |
| CpG_11.12 | 4_583 | 5_444 | 0.000 | 0.0272 | -0.0699 | 0.0699 | 1.0000 |
| CpG_14 | 2_580 | 5_444 | 0.060 | 0.0297 | -0.0163 | 0.1363 | 0.0990 |
| CpG_14 | 2_580 | 1_438 | 0.050 | 0.0297 | -0.0263 | 0.1263 | 0.1527 |
| CpG_14 | 3_435 | 5_444 | 0.045 | 0.0297 | -0.0313 | 0.1213 | 0.1897 |
| CpG_14 | 4_583 | 5_444 | 0.040 | 0.0297 | -0.0363 | 0.1163 | 0.2354 |
| CpG_14 | 3_435 | 1_438 | 0.035 | 0.0297 | -0.0413 | 0.1113 | 0.2911 |
| CpG_14 | 4_583 | 1_438 | 0.030 | 0.0297 | -0.0463 | 0.1063 | 0.3583 |
| CpG_14 | 2_580 | 4_583 | 0.020 | 0.0297 | -0.0563 | 0.0963 | 0.5301 |
| CpG_14 | 2_580 | 3_435 | 0.015 | 0.0297 | -0.0613 | 0.0913 | 0.6346 |
| CpG_14 | 1_438 | 5_444 | 0.010 | 0.0297 | -0.0663 | 0.0863 | 0.7497 |
| CpG_14 | 3_435 | 4_583 | 0.005 | 0.0297 | -0.0713 | 0.0813 | 0.8728 |
| CpG_15 | 2_580 | 3_435 | 0.055 | 0.0237 | -0.0058 | 0.1158 | 0.0677 |
| CpG_15 | 2_580 | 5_444 | 0.050 | 0.0237 | -0.0108 | 0.1108 | 0.0883 |
| CpG_15 | 2_580 | 4_583 | 0.050 | 0.0237 | -0.0108 | 0.1108 | 0.0883 |
| CpG_15 | 2_580 | 1_438 | 0.040 | 0.0237 | -0.0208 | 0.1008 | 0.1518 |
| CpG_15 | 1_438 | 3_435 | 0.015 | 0.0237 | -0.0458 | 0.0758 | 0.5540 |
| CpG_15 | 1_438 | 5_444 | 0.010 | 0.0237 | -0.0508 | 0.0708 | 0.6902 |
| CpG_15 | 1_438 | 4_583 | 0.010 | 0.0237 | -0.0508 | 0.0708 | 0.6902 |
| CpG_15 | 4_583 | 3_435 | 0.005 | 0.0237 | -0.0558 | 0.0658 | 0.8410 |
| CpG_15 | 5_444 | 3_435 | 0.005 | 0.0237 | -0.0558 | 0.0658 | 0.8410 |
| CpG_15 | 4_583 | 5_444 | 0.000 | 0.0237 | -0.0608 | 0.0608 | 1.0000 |
| CpG_16 | 2_580 | 3_435 | 0.060 | 0.0045 | 0.0485 | 0.0715 | <.0001 |
| CpG_16 | 2_580 | 5_444 | 0.060 | 0.0045 | 0.0485 | 0.0715 | <.0001 |
| CpG_16 | 2_580 | 1_438 | 0.045 | 0.0045 | 0.0335 | 0.0565 | 0.0002 |
| CpG_16 | 2_580 | 4_583 | 0.045 | 0.0045 | 0.0335 | 0.0565 | 0.0002 |
| CpG_16 | 1_438 | 3_435 | 0.015 | 0.0045 | 0.0035 | 0.0265 | 0.0202 |
| CpG_16 | 4_583 | 3_435 | 0.015 | 0.0045 | 0.0035 | 0.0265 | 0.0202 |
| CpG_16 | 1_438 | 5_444 | 0.015 | 0.0045 | 0.0035 | 0.0265 | 0.0202 |
| CpG_16 | 4_583 | 5_444 | 0.015 | 0.0045 | 0.0035 | 0.0265 | 0.0202 |
| CpG_16 | 4_583 | 1_438 | 0.000 | 0.0045 | -0.0115 | 0.0115 | 1.0000 |
| CpG_16 | 5_444 | 3_435 | 0.000 | 0.0045 | -0.0115 | 0.0115 | 1.0000 |
| Average | 1_438 | 3_435 | 0.139 | 0.0187 | 0.0907 | 0.1868 | 0.0007 |
| Average | 1_438 | 4_583 | 0.135 | 0.0187 | 0.0865 | 0.1826 | 0.0008 |
| Average | 1_438 | 5_444 | 0.131 | 0.0187 | 0.0832 | 0.1793 | 0.0009 |
| Average | 2_580 | 3_435 | 0.130 | 0.0187 | 0.0815 | 0.1776 | 0.0010 |
| Average | 2_580 | 4_583 | 0.125 | 0.0187 | 0.0774 | 0.1735 | 0.0011 |
| Average | 2_580 | 5_444 | 0.122 | 0.0187 | 0.0740 | 0.1701 | 0.0013 |
| Average | 1_438 | 2_580 | 0.009 | 0.0187 | -0.0389 | 0.0572 | 0.6447 |
| Average | 5_444 | 3_435 | 0.008 | 0.0187 | -0.0406 | 0.0556 | 0.7049 |
| Average | 4_583 | 3_435 | 0.004 | 0.0187 | -0.0439 | 0.0522 | 0.8325 |
| Average | 5_444 | 4_583 | 0.003 | 0.0187 | -0.0447 | 0.0514 | 0.8655 |
