# Supplementary figures and images for: Stable oncogenic silencing in vivo by programmable and targeted de novo DNA methylation in breast cancer
Source: Oncogene. 2015 Feb 16;34(43):5427–35. doi: 10.1038/onc.2014.470 (PMC4633433; doi:10.1038/onc.2014.470)

Figure S1

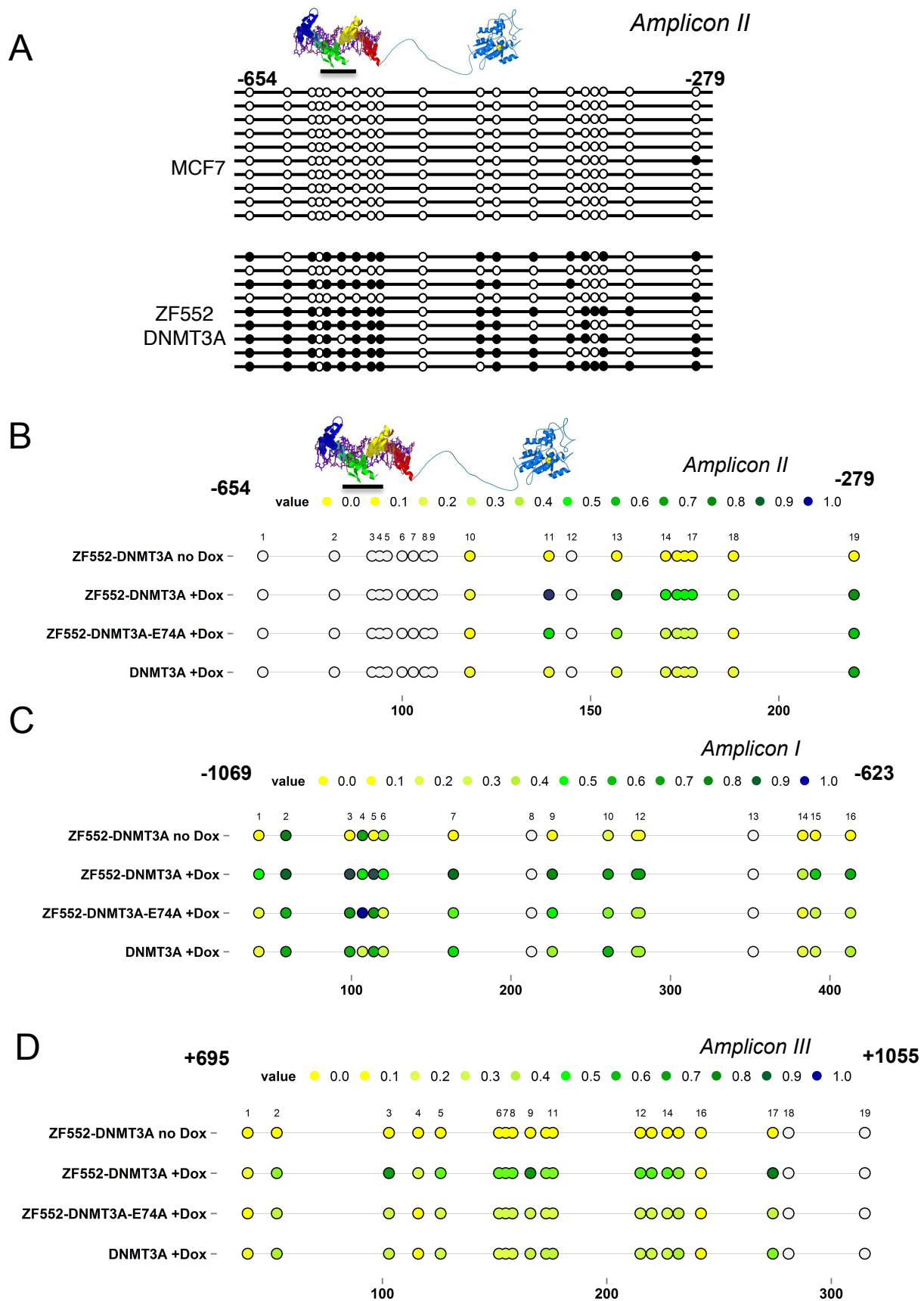

Supplement: Supplementary Figure S1 [file onc2014470x2.pdf]

Supplemental Figure S2

*MASPIN PROMOTER*

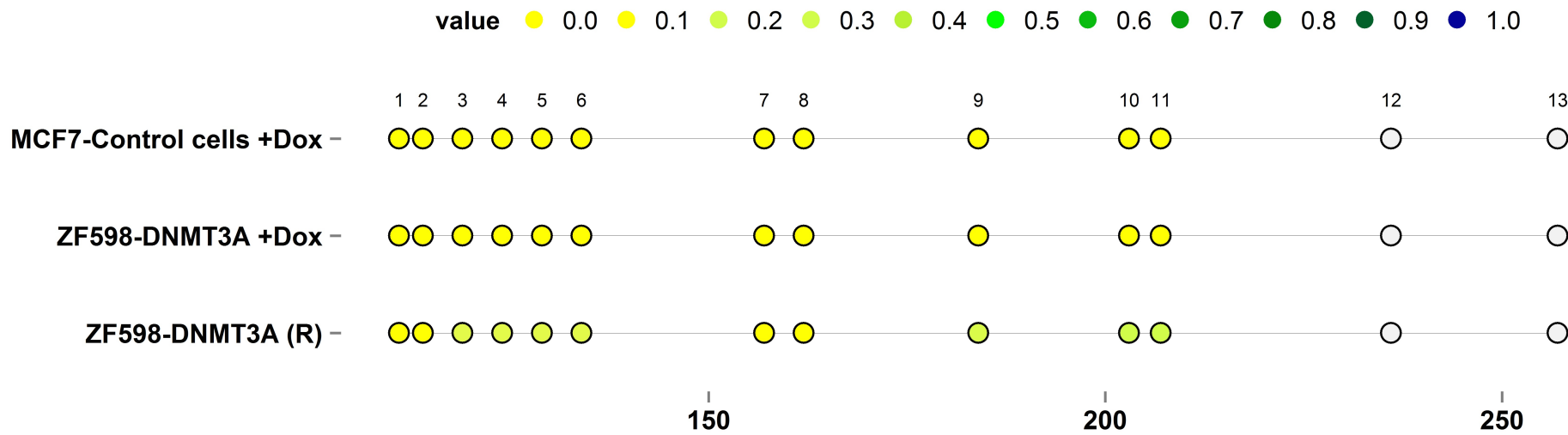

Supplement: Supplementary Figure S2 [file onc2014470x3.pdf]

A Supplemental Figure S3

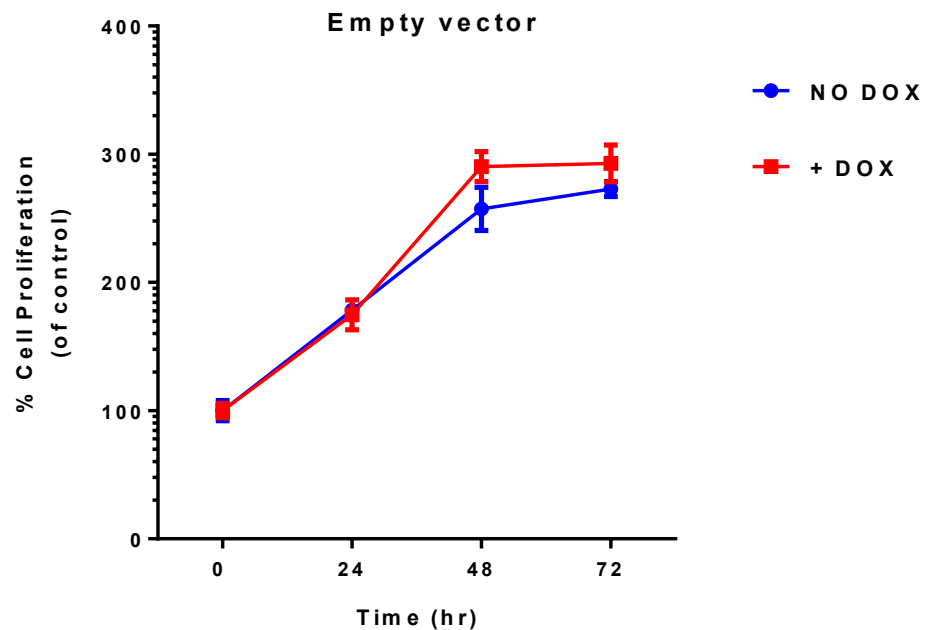

B

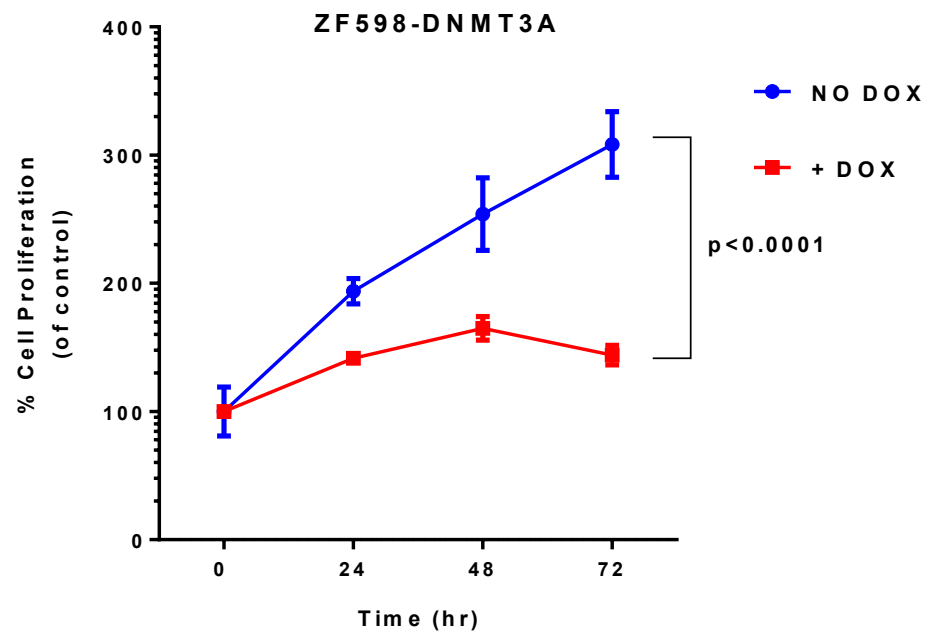

C

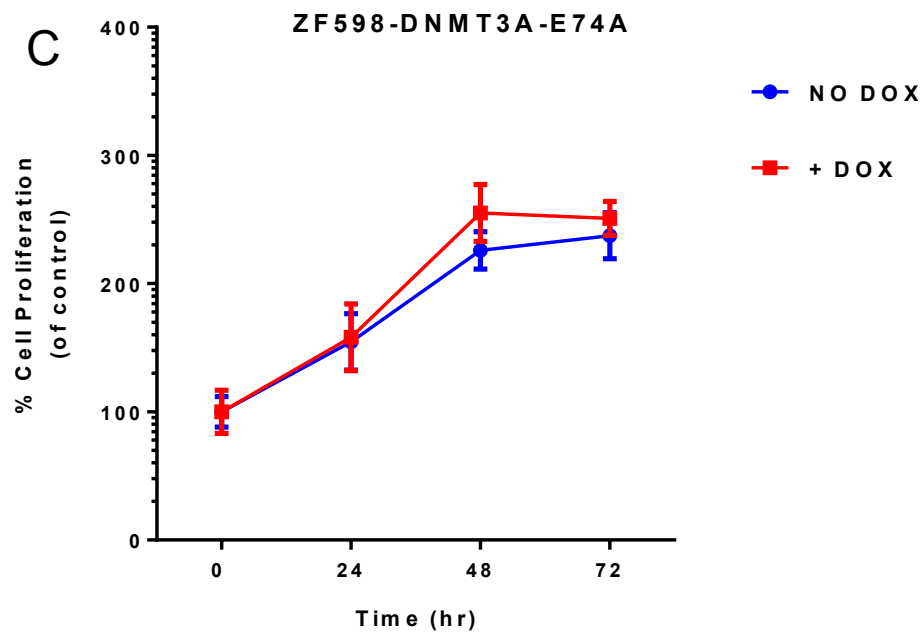

Supplement: Supplementary Figure S3 [file onc2014470x4.pdf]

Supplemental Figure S5

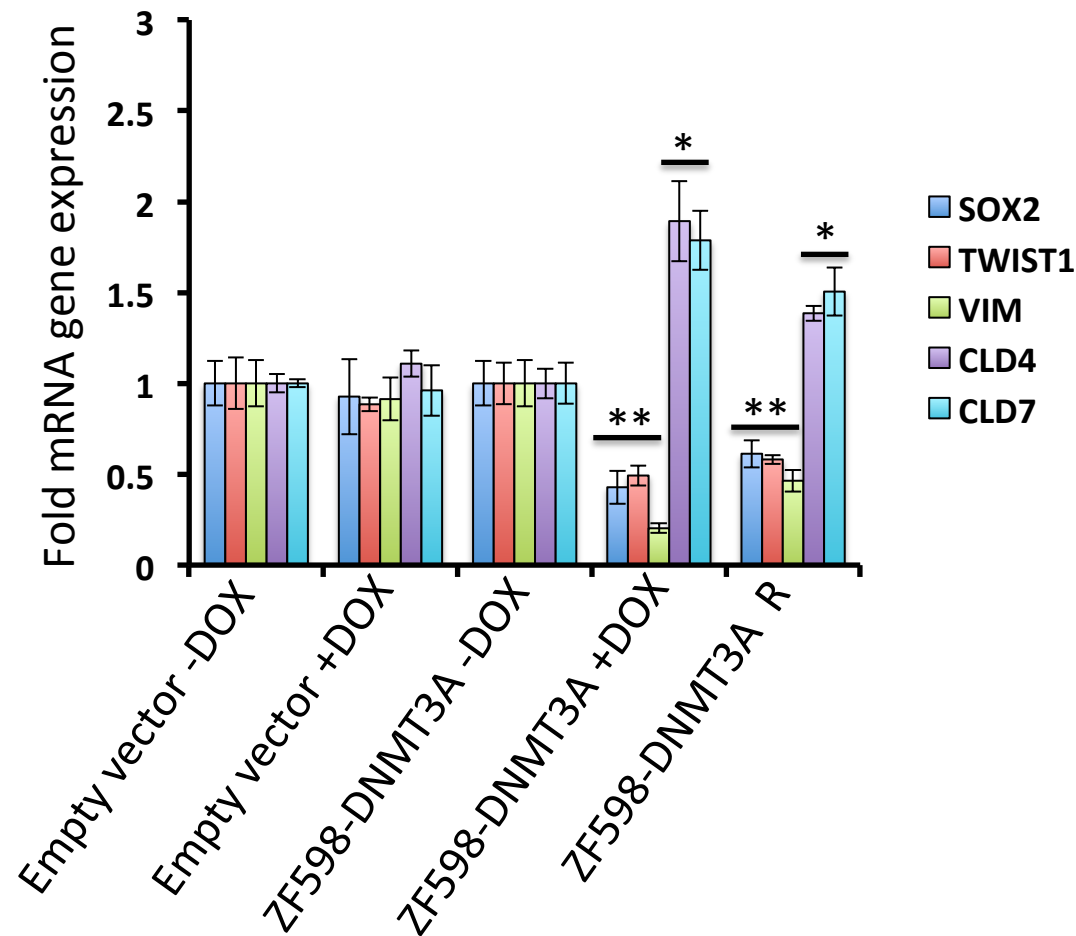

Supplement: Supplementary Figure S5 [file onc2014470x6.pdf]
