# Supplementary material for: Stable oncogenic silencing in vivo by programmable and targeted de novo DNA methylation in breast cancer
Source: Oncogene. 2015 Feb 16;34(43):5427–35. doi: 10.1038/onc.2014.470 (PMC4633433; doi:10.1038/onc.2014.470)
Supplement: Supplementary Figure S4 [file onc2014470x5.pdf]

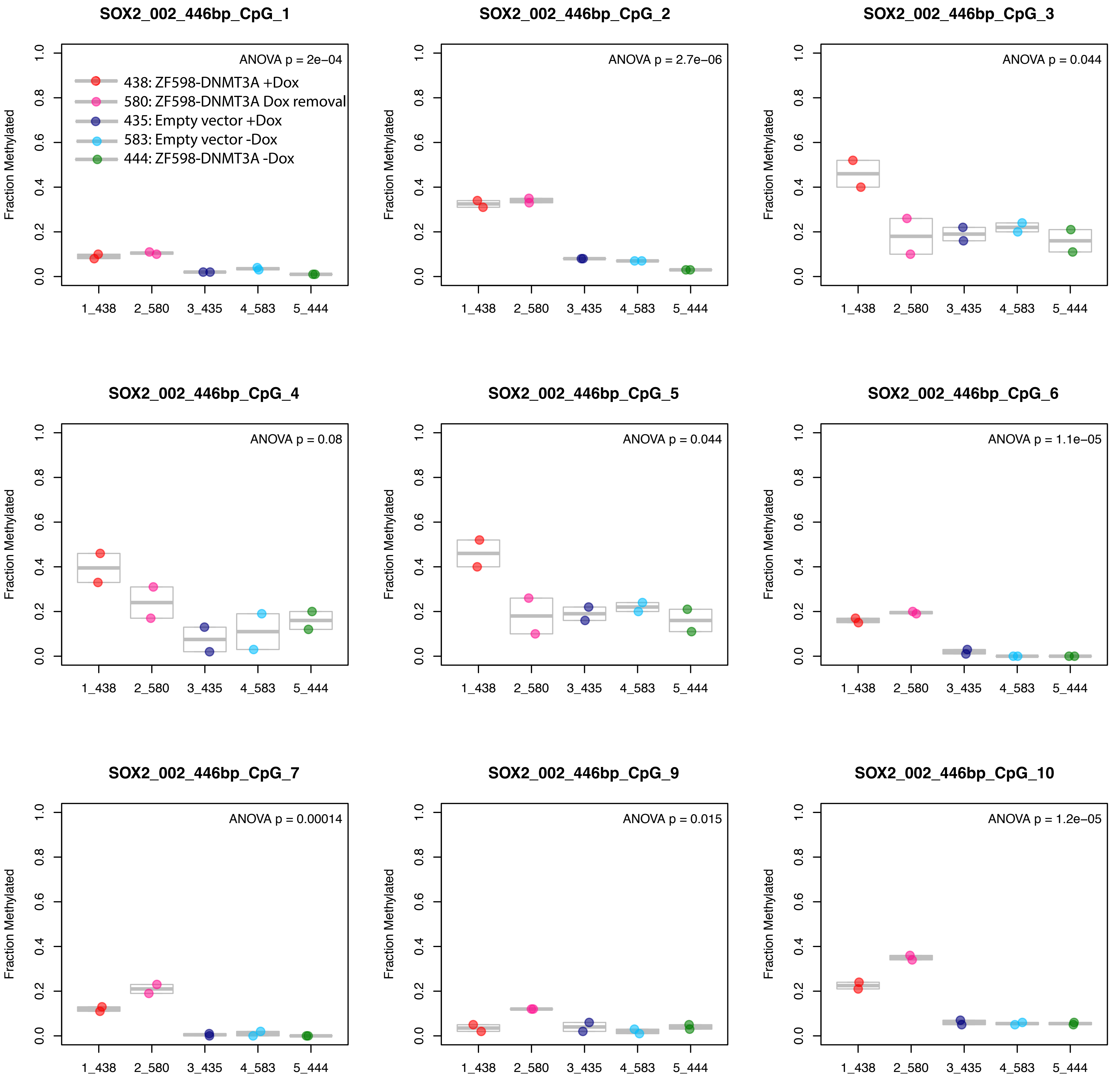

SOX2_002_446bp_CpG_1
ANOVA p = 2e−04
438: ZF598-DNMT3A +Dox
580: ZF598-DNMT3A Dox removal
435: Empty vector +Dox
583: Empty vector -Dox
444: ZF598-DNMT3A -Dox
SOX2_002_446bp_CpG_2
ANOVA p = 2.7e−06
SOX2_002_446bp_CpG_3
ANOVA p = 0.044
SOX2_002_446bp_CpG_4
ANOVA p = 0.08
SOX2_002_446bp_CpG_5
ANOVA p = 0.044
SOX2_002_446bp_CpG_6
ANOVA p = 1.1e−05
SOX2_002_446bp_CpG_7
ANOVA p = 0.00014
SOX2_002_446bp_CpG_9
ANOVA p = 0.015
SOX2_002_446bp_CpG_10
ANOVA p = 1.2e−05
Fraction Methylated
0.0
0.2
0.4
0.6
0.8
1.0
1_438
2_580
3_435
4_583
5_444

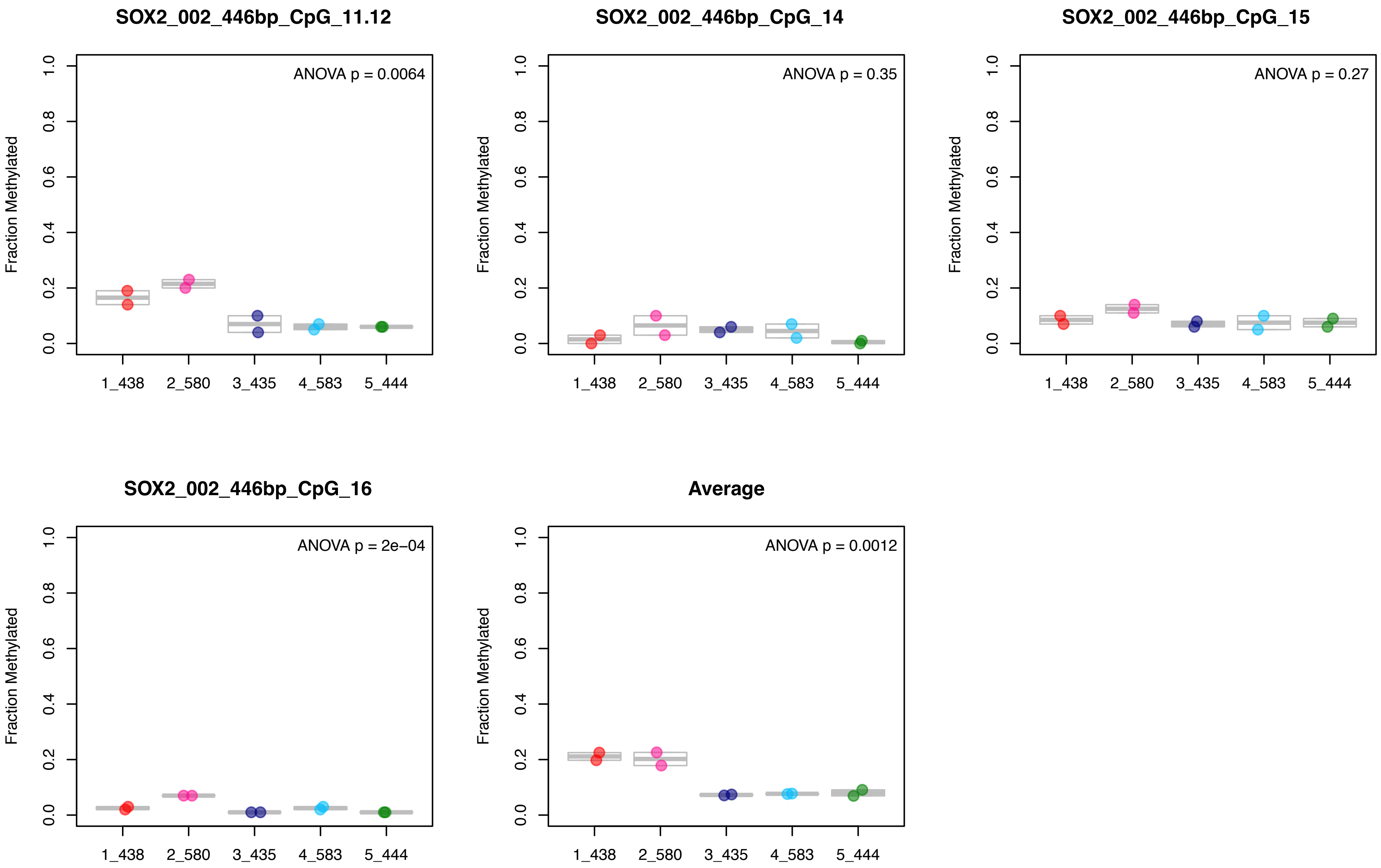
SOX2_002_446bp_CpG_11.12
ANOVA p = 0.0064
SOX2_002_446bp_CpG_14
ANOVA p = 0.35
SOX2_002_446bp_CpG_15
ANOVA p = 0.27
SOX2_002_446bp_CpG_16
ANOVA p = 2e−04
Average
ANOVA p = 0.0012
Fraction Methylated
0.0
0.2
0.4
0.6
0.8
1.0
1_438
2_580
3_435
4_583
5_444
