# Supplementary material for: Stable oncogenic silencing in vivo by programmable and targeted de novo DNA methylation in breast cancer
Source: Oncogene. 2015 Feb 16;34(43):5427–35. doi: 10.1038/onc.2014.470 (PMC4633433; doi:10.1038/onc.2014.470)
Supplement: Supplementary Figure Legends [file onc2014470x7.doc]

**Supplementary figure legends**

**Supplementary Figure S1:** **Expression of a second ZF-DNMT3A fusion protein (ZF552-DNMT3A) induces targeted DNA methylation in the *SOX2* promoter.** (**a)** Sodium bisulfite sequencing analysis of DNA derived from MCF7 cells stably transduced with empty vector, ZF552-DNMT3A and ZF552-DNMT3A-E74A and induced with Dox. The analyzed amplicon II (-654 to -279 base pairs (bps) is located upstream of the translation start site and comprises the 6ZF binding site (position -552 relative to the translation start site). **(b)** MassARRAY analysis of amplicon II (same as in **(a)**). Circles indicate the CpG dinucleotides in the amplicon (Color-code: yellow = unmethylated CpG to blue = 100% methylated CpG). Grey circles indicate not analyzed methylation values due to CpGs with high or low mass Dalton peaks falling outside the conservative window of reliable detection for the EpiTYPER software. **(c)** MassARRAY analysis of amplicon I (-1069 to -623 bps upstream of the translation start site) after Dox-induction. **(d)** MassARRAY analysis of amplicon III (+695 to +1055 bps) downstream of the translation start site after Dox-induction.

**Supplementary Figure S2: Expression of ZF598-DNMT3A in MCF7 cells does not alter *MASPIN* promoter methylation.** MassARRAY analysis of the proximal MASPIN promoter outlining the 13 CpG nucleotides in MCF7 cells[^19^](#_ENREF_19). Circles indicate the CpG dinucleotides in the amplicon (Color-code: yellow = unmethylated CpG to blue = 100% methylated CpG). Grey circles indicate not analyzed methylation values due to CpGs with high or low mass Dalton peaks falling outside the conservative window of reliable detection for the EpiTYPER software.

**Supplementary Figure S3: Induction of expression of ZF598-DNMT3E74A by Dox does not significantly alter tumor cell growth viability.** Cells were maintained in -Dox conditions or induced with Dox as described above and the tumor cell viability was monitored with a [CellTiter-Glo®](http://www.promega.com/products/pm/celltiter-glo-20/) assay.

**Supplementary Figure S4: Analysis of tumor xenografts shows increased CpG methylation upon induction and removal of ZF598-DNMT3A expression at 43 days post-induction.** Sequenom EpiTYPER MassARRAY platform was used to quantify percent methylation for CpG dinucleotides within amplicon I (annotated as SOX2_002 in the Figure S4) in the tumors retrieved from mice with the following five treatments: 1. Red, tumor 438: ZF598-DNMT3A +Dox (43 days post-induction). 2. Pink, tumor 580: 598-ZFDNMT3A Dox removal, 43 days post-induction. 3. Dark blue, 435 tumor: Empty vector +Dox, 29 days post-induction. 4. Light blue, tumor 583: Empty vector -Dox, 29 days post-induction and 5. Green, tumor 444: ZF598-DNMT3A -Dox, 29 days post-induction. By ANOVA and students t-test, differences between treatment groups for the majority of CpG units interrogated were statistically significant at *p* values ranging from <0.04 to <0.002 (Supplemental Table S1).

**Supplementary Figure S5: RNA extraction from tumor xenografts reveals changes in expression of mesenchymal to epithelial transition (MET) markers.** RNA was extracted from the tumor xenografts generated from empty vector control cells at day 29 post-induction (tumor 581 (-Dox) and tumor 435 (+Dox)) and from ZF598-DNMT3A xenografts at day 43 post-induction (tumor 436 (-Dox), tumor 598 (+Dox) and tumor 589 (Dox removal)). mRNA marker expression was quantitatively analyzed by taqman qRT-PCR and data normalized to empty vector control -Dox. Significant changes in gene expression upon Dox induction and Dox removal in four replicates were investigated with a student t test (***p*<0.05; **p*<0.01).
